# Adjunctive electrophysical therapies used in addition to land-based exercise therapy for osteoarthritis of the hip or knee: A systematic review and meta-analysis

**DOI:** 10.1016/j.ocarto.2024.100457

**Published:** 2024-03-01

**Authors:** Helen P. French, Joice Cunningham, Rose Galvin, Sania Almousa

**Affiliations:** a School of Physiotherapy, Royal College of Surgeons in Ireland (RCSI) University of Medicine and Health Sciences, Dublin 2, Ireland; b School of Allied Health, University of Limerick, Limerick, Ireland

**Keywords:** Systematic review, Physical therapies, Exercise therapy, Osteoarthritis

## Abstract

**Objectives:**

To review evidence for effectiveness of electrophysical therapies (EPTs), used adjunctively with land-based exercise therapy, for hip or knee osteoarthritis (OA), compared with 1) placebo EPTs delivered with land-based exercise therapy or 2) land-based exercise therapy only.

**Methods:**

Six databases were searched up to October 2023 for randomised controlled trials (RCTs)/quasi-RCTs comparing adjunctive EPTs alongside land-based exercise therapy versus 1) placebo EPTs alongside land-based exercise, or 2) land-based exercise in hip or knee OA. Outcomes included pain, function, quality of life, global assessment and adverse events. Risk of bias and overall certainty of evidence were assessed. We back-translated significant Standardised Mean Differences (SMDs) to common scales: 2 points/15% on a 0–10 Numerical Pain Rating Scale and 6 points/15% on the WOMAC physical function subscale.

**Results:**

Forty studies (2831 patients) evaluated nine different EPTs for knee OA. Medium-term effects (up to 6 months) were evaluated in seven trials, and one evaluated long-term effects (>6 months). Adverse events were reported in one trial. Adjunctive laser therapy may confer short-term effects on pain (SMD -0.68, 95%CI -1.03 to −0.34; mean difference (MD) 1.18 points (95% CI -1.78 to −0.59) and physical function (SMD -0.60, 95%CI -0.88 to −0.34; MD 12.95 (95%CI -20.05 to −5.86)) compared to placebo EPTs, based on very low-certainty evidence. No other EPTs (TENS, interferential, heat, shockwave, shortwave, ultrasound, EMG biofeedback, NMES) showed clinically significant effects compared to placebo/exercise, or exercise only.

**Conclusions:**

Very low-certainty evidence supports laser therapy used adjunctively with exercise for short-term improvement in pain and function. No other EPTs demonstrated clinically meaningful effects.

## 1 Introduction

Non-pharmacological interventions including exercise, education and weight management are recommended core treatments for osteoarthritis (OA) across numerous clinical guidelines [[1], [2], [3], [4]]. Other conservative non-pharmacological therapies combined with exercise therapy include electrophysical therapies (EPTs), which deliver thermal, mechanical, light, sound or electrical energy to provide physiological changes and confer therapeutic effects. EPTs are largely not recommended in international OA clinical guidelines due to low quality evidence or implausible biological mechanisms [[1], [2], [3], [4]], although thermal agents and ultrasound were conditionally recommended in one guideline [2].

A Cochrane review of 62 trials (6508 participants) evaluating the effects of adjunctive non-pharmacological therapies used with land-based exercise therapy found, based on moderate-to low-certainty evidence, no difference in pain, physical function or quality of life (QoL) between adjunctive EPTs compared to their placebo equivalent/exercise, or exercise only [5]. When various EPTs, such as therapeutic ultrasound (US), laser therapy, transcutaneous electrical nerve stimulation (TENS), shortwave/pulsed electromagnetic energy (PEME), interferential therapy (IFT), electromyographic (EMG) biofeedback and shockwave therapy were combined together into one adjunctive therapy EPT subgroup for analysis, we found no important effects across outcomes in either comparison (adjunctive placebo therapy/exercise or exercise) [5]. However, as the effects of specific EPTs were not investigated, further exploration of their individual effects when used as adjuncts to land-based exercise for hip or knee OA was warranted.

This review aimed to compare the effects of different types of EPTs, used adjunctively with land-based exercise therapy, for people with hip or OA, compared with 1) a placebo EPTs delivered with land-based exercise therapy or 2) land-based exercise therapy only.

## 2 Method

This review protocol was prospectively registered with PROSPERO (CRD-42022380247) and reported in accordance with the Preferred Reporting Items for Systematic Reviews and Meta-Analyses (PRISMA) guidelines [6].

### 2.1 Search strategy and screening

We updated searches completed for the Cochrane review (up to June 2021), by searching the following databases, retaining all search terms from the original review, up to 26 Oct 2023: Medline, Cochrane Central Register of Controlled Trials, PsycINFO, EMBASE, CINAHL Plus and the Physiotherapy Evidence Database (https://pedro.org.au/), with no language restrictions (supplemental file 1). Two review authors (HPF and SA) independently screened titles and abstracts of retrieved citations from the updated searches against the review eligibility criteria, following removal of duplicates. Full-texts were retrieved, when eligibility could not be determined from the title or abstract. Screening was undertaken using Covidence (Veritas Health Innovation, Melbourne, Australia).

### 2.2 Eligibility criteria

Our inclusion criteria were Randomised Controlled Trials (RCTs) or quasi-RCTs, adults aged ≥18 years, with clinical or radiographic diagnosis of hip or knee OA as defined in the trials. Where trials included mixed populations, at least 75% of participants must have had knee and/or hip OA [7]. The intervention was adjunctive EPTs, which could include, but were not limited to thermal modalities, therapeutic US, laser therapy, TENS, pulsed electromagnetic energy (PEME)/shortwave, interferential therapy (IFT), electromyographic (EMG) biofeedback, phonophoresis, iontophoresis or shockwave therapy, delivered with land-based exercise. The comparator intervention was land-based exercise therapy, which could include supervised or home-based exercise regimens for knee or hip OA, delivered with a placebo adjunctive EPT or on its own. The exercise therapy in both groups must have been identical so that the only difference between the groups was the addition of the EPT. Comparators could include either 1) the same placebo adjunctive EPT delivered with identical exercise therapy, or 2) identical exercise therapy, with no adjunctive EPT. Abstracts, protocols, conference proceedings and unpublished data were excluded.

### 2.3 Outcomes

We included studies assessing pain and physical function as the primary outcomes [8]. Secondary outcomes included participant-reported global assessment, QoL, radiographic joint structure changes and adverse events.

All outcomes were assessed immediately post-intervention (short-term). If intervention duration differed between exercise therapy and adjunctive EPT, the EPT time-point was used. We also assessed medium-term (<six months) and long-term outcomes (>six months post-intervention).

### 2.4 Data extraction

Data extraction for the Cochrane review was undertaken by two independent review authors (HPF/RG), and by HPF and JC for additional studies identified up to 2023. We extracted follow-up data, with related standard deviations (SDs) for continuous outcomes. Missing SDs were calculated using provided 95% confidence intervals (CIs). If further data were required, study authors were contacted a maximum of three times via email. We used graph digitisation software (https://automeris.io/WebPlotDigitizer/) to extrapolate means and SDs by digitalising data points on graphs in two studies [9,11].

### 2.5 Risk of bias assessment

Risk of bias (RoB) of the six new studies was assessed by three independent review authors (HPF, SA and JC) using the Cochrane RoB V1 tool, while retaining original RoB judgements from the Cochrane review for this review [5]. We graded each source of bias as low, high or unclear risk and discussed RoB judgements to reach consensus.

### 2.6 Data synthesis and analysis

Cochrane's Review Manager (version 5.4) was used for data analysis, using a random-effects model, assuming clinical heterogeneity. Dichotomous outcomes (e.g. adverse events) were reported as risk ratios (RRs) with 95% CIs. Continuous outcomes (pain, function, QoL), were calculated using Standardised Mean Differences (SMDs) and 95% CIs, using post-intervention values and if not available, we used change scores [10].

Statistical heterogeneity was assessed using the I^2^ statistic (0%–40%: might not be important, 30%–60%: moderate heterogeneity, 50%–90%: substantial heterogeneity, 75%–100%: considerable heterogeneity) [12]. Subgroup analysis of different EPTs was undertaken in line with our research question, as well as overall treatment effects.

Where pooled effect estimates of continuous outcomes were statistically significant, to enhance interpretability of measures of treatment effect, we back-translated SMDs to a common scale by multiplying the SMD by a typical among-person standard deviation (SD) [13]. This was obtained from a control group SD from the most representative trial with the highest weight in the meta-analysis and least susceptibility to bias. For pain measures, we back-translated to an 11-point Numerical Pain Rating Scales (NPRS), assuming that visual analogue scales (VAS) and NPRS were comparable. For physical function, we back-translated to the Likert version (0-68) WOMAC physical function subscale [14]. We assumed a minimal clinically important difference (MCID), including mean difference and 95% CIs of 2 points [15] or relative (percentage) difference of 15% [16] on a 0–10 NPRS, 6 points on the 0–68 WOMAC Likert physical function subscale [17], and percentage change of 15%. Further detail on data synthesis and analysis is available in the Cochrane review [5].

Due to addition of new trials in this review, the Grading of Recommendations, Development and Evaluation (GRADE) of the overall certainty of the evidence in each pooled analysis from the Cochrane review was reassessed by two independent review authors (HPF and SA or JC), using GRADEPro software. Domains assessed included RoB, consistency of effect, precision of effect estimates, directness of the evidence and publication bias [18]. Certainty of cumulative evidence started at ‘high’ and was downgraded for each domain not judged as low risk (Supplemental file 2).

## 3 Results

### 3.1 Study selection

**Fig. 1:**
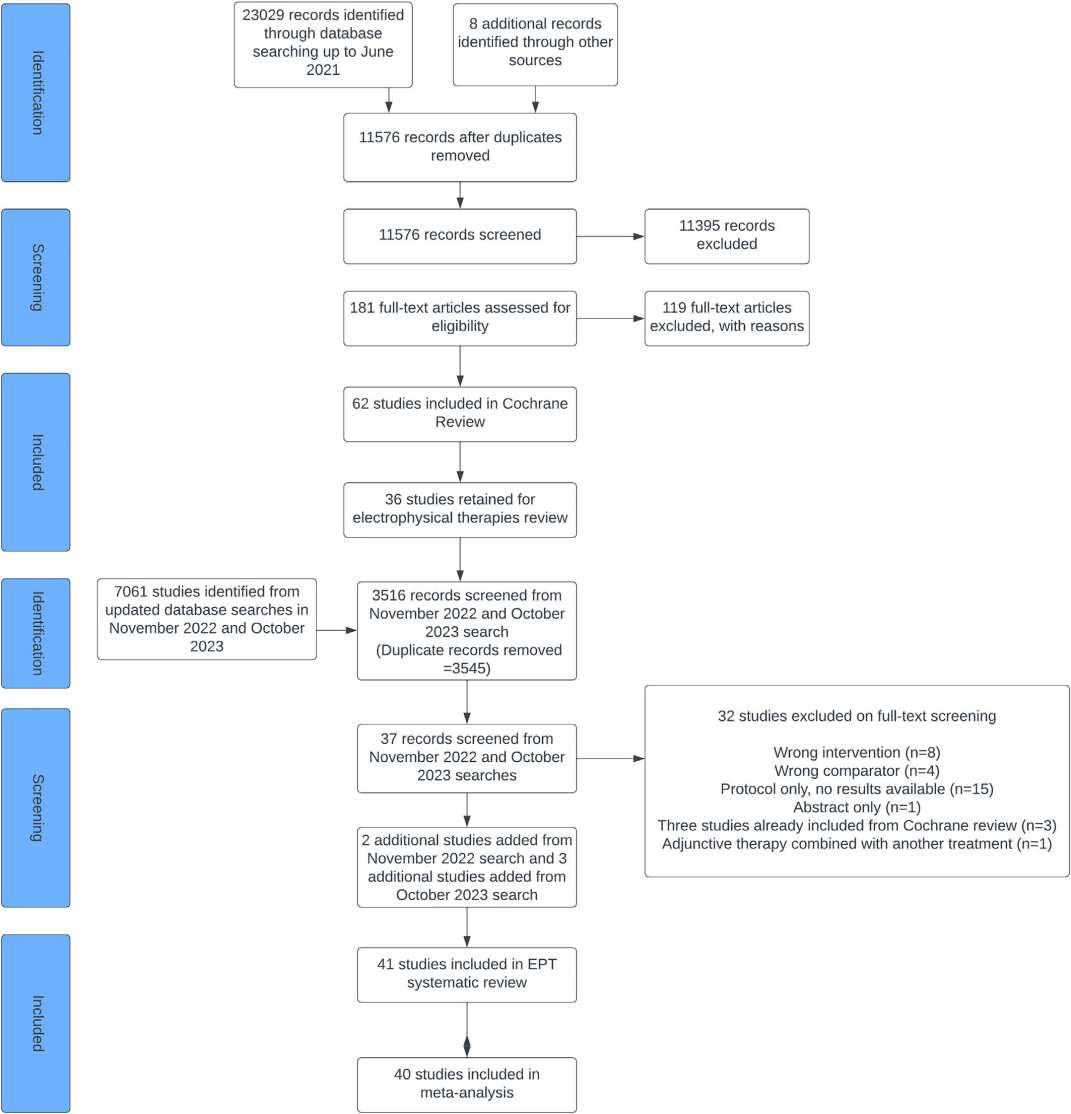
PRISMA flow diagram.

The original Cochrane review search conducted up to 10 June 2021 identified 181 articles, which were retained for full-text screening, from which 62 studies were included. For this review, 36 studies, which investigated adjunctive EPTs were retained, and searches (using the original search strategy (Supplementary file 1) rerun across the six databases in November 2022 and updated in October 2023. Following screening of 3516 titles and abstracts by two review authors (HPF and SA), 37 were retained for full-text screening and six studies were included in this review [11,[19], [20], [21], [22], [23]], along with the original 36 EPT trials from the Cochrane review [5]. Of these newly included studies, one reported different outcomes [21] from a trial previously included in the Cochrane review [24]. Therefore, 41 papers from 40 trials (2831 participants) were included in this review. The PRISMA flow diagram is shown in Fig. 1.

### 3.2 Study characteristics

All studies, except one, which was a quasi-randomised trial [25], were RCTs and all trials employed a parallel group design. Nineteen studies compared the adjunctive EPT plus exercise therapy to a placebo EPT and exercise [[22], [23], [24], [25], [26], [27], [28], [29], [30], [31], [33], [34], [35], [36], [37], [38], [39], [40], [41]], whilst 22 compared against exercise only [9,11,19,20,22,[34], [35], [42], [43], [44], [45], [46], [47], [48], [49], [50], [51], [52], [53], [54], [55], [57]].

Seventeen trials compared two groups [11,23,25,[28], [29], [30],^33^,^36^,^39^,^41^,[43], [44], [45],^48^,^49^,^54^], 15 included three groups [9,19,20,22,27,31,34,37,38,40,46,47,51,55,56], five compared four groups [21,24,42,50,52], one compared five groups [57] and one trial had six different arms, comparing three EPTs to placebo counterparts [26]. The number of groups eligible for inclusion is shown in Table 1, with two groups included in 27 studies [11,19,20,[23], [24], [25],^28^,^30^,^33^,^36^,^39^,[41], [42], [43], [44], [45], [46], [47], [48], [49], [50], [51],^53^,^54^,^57^,^58^], three groups in 12 studies [9,22,27,31,34,37,38,40,55,56,59,60], one study each including four [52] or six groups [26]. Studies took place in 16 countries (Table 1). Five studies included women only [19,20,33,57,61], one recruited only men [38], with the remainder recruiting both. Total sample size varied from 20 to 203, with sample size per group ranging from 20 to 60 participants. Sample size estimates were provided in 21/40 trials (53%), with varying details. All studies recruited people with knee OA. Mean age ranged from 52 to 69 years (Table 1).

**Table 1:** Characteristics of included studies.

Author year	Country	Groups eligible for inclusion in the review	Number per group	Mean age (SD))	Adjunctive Therapy	Placebo	Intervention length	Follow-up	Outcomes
Adedoyin 2002	Nigeria	G1: IFT ​+ ​Ex G2: Placebo IFT ​+ ​Ex	15 per group	G1: 60 (2.3) G2: 58.4 (3.6)	IFT	Yes	4 weeks	4 weeks	VAS Pain
Adedoyin 2005	Nigeria	G1: TENS ​+ ​Ex G2: IFT ​+ ​Ex G3: Ex	G1: 15 G2: 19 G3: 17	G1: 55.40 (9.55) G2: 52.30 (11.03) G3: 56.87 (6.53)	TENSIFT	No	4 weeks	weeks 1, 2, 3, 4:	WOMAC Total score 10-point pain intensity scale
Adhya 2015	India	GI: PEME ​+ ​Ex G2: IFT ​+ ​Ex G3: US ​+ ​Ex G4 Ex	50 per group	NR	PEME IFT US	No	8 weeks	8 weeks	VAS Pain WOMAC total score
Akaltun 2021	Turkey	G1: HILT ​+ ​Ex G2: Placebo HILT ​+ ​Ex	20 per group	G1: 57.8 (8.1) G2: 58.6 (11.3)	Laser	Yes	2 weeks	2 weeks and 6 weeks	VAS Pain WOMAC Flexion ROM Femoral cartilage thickness
Akyol 2010	Turkey	G1: SWD ​+ ​Ex G2: Ex	20 per group	G1: 57.80 (10.65) G2: 65.60 (8.13)	SWD	No	4 weeks	4 weeks and 3 months	VAS pain WOMAC SF-36
Alfredo 2018	Brazil	G1: Laser ​+ ​Ex G2: Placebo laser ​+ ​Ex	G1: 24 G2: 22	G1: 61.15 (7.52) G2: 62.25 (6.87)	Laser	Yes	3 weeks of laser, followed by 8 weeks of exercise	3, 11, 24 and 37 weeks	VAS Pain WOMAC Lequesne Index Knee ROM Isometric quads strength
Alghadir 2014	Saudi Arabia	G1: Laser ​+ ​Ex G2: Placebo laser ​+ ​Ex	20 per group	G1: 55.2 (8.14) G2: 57 (7.77)	Laser	Yes	4 weeks	4 weeks	VAS Pain WOMAC 50-foot walk
Al-Rashoud 2014	Saudi Arabia	G1: Laser ​+ ​Ex G2: Placebo laser ​+ ​Ex	G1: 30 G2: 28	G1: 52 (9) G2: 55 (11)	Laser	Yes		3 and 11 weeks	VAS Pain Saudi Knee Function Scale Active knee flexion Patient Satisfaction
Atamaz 2012	Turkey	G1: TENS ​+ ​Ex G2: IFT ​+ ​Ex G3: SWD ​+ ​Ex G4: Placebo TENS ​+ ​Ex G5: Placebo IFT ​+ ​Ex G6: Placebo SWD ​+ ​Ex	G1: 37 G2: 31 G3:31 G4:37 G5:35 G6:32	G1: 61.9 (6.9) G2: 62 (7.9) G3: 61.4 (8.2) G4: 60.7 (6.5) G5: 61.3 (7.8) G6: 61.54 (8.2)	TENS SWD IFT	Yes	3 weeks	1, 3 and 6 months	VAS Pain WOMAC
Cakir 2014	Turkey	G1: Continuous US ​+ ​Ex G2: Pulsed US ​+ ​Ex G3: Placebo US ​+ ​Ex	20 per group	G1: 56.9 (8.8) G2: 58.2 (9.9) G3: 57.1 (7.8)	Continuous US Pulsed US	Yes	3 weeks	2 weeks, 6 months	VAS Pain WOMAC 20 ​m walk test VAS disease severity
Carlos 2012	Brazil	G1: Continuous US ​+ ​Ex G2: Pulsed US ​+ ​Ex G3: Ex	10 per group	G1: 63.9 (6.31) G2: 63.4 (4.59) G3: 62.7 (8.71)	Continuous US Pulsed US	No	US- 4 weeks Exercise −8 weeks	Post-treatment	WOMAC Knee ROM TUG Quads strength
Cetin 2008	Turkey	G4: Hot packs ​+ ​Ex G5: Ex	20 per group	G4: 61.05 (8.26) G5: 58.85 (9.08)	Hot pack was provided prior to exercise	No	3 times a week for 8 weeks	8 weeks	VAS pain, Lequesne Index of Severity, isokinetic strength, 50 ​m walk test
Cheing 2004	Hong Kong	G1: TENS ​+ ​Ex G2: Ex	G1: 17 G2: 15	G1: 64.3 (9.2) G2: 60.9 (7.3)	TENS	No	4 weeks	Sessions 1, 10, 20; 4 weeks	VAS Pain
Chen 2014	Taiwan	G1: Shockwave ​+ ​Ex G2: Pulsed US ​+ ​Ex G3: Ex	30 per group	Mean age: 63.0 (7.4)	Shockwave US	No	8 weeks	8 weeks and 6 months	VAS Pain Lequesne Index
De Matos Brunelli Braghin 2018	Brazil	G1: Laser ​+ ​Ex G2: Ex	28 per group	G1: 64.6 (5.24) G2: 58.57 (7.42)	Laser	No	2 months	2 months	WOMAC
de Paula Gomes 2018	Brazil	G1: Laser ​+ ​Ex G2: Placebo Laser ​+ ​Ex G3: Ex	20 per group	G1: 65.15 (4.90) G2: 67.20 (3.95) G3: 64.75 (3.76)	Laser	Yes	5 weeks	5 weeks	NPRS Pain WOMAC LEFS PPT FRT Isometric strength (GMed and Quads)
Elboim-Gabyzon 2013	Israel	G1: NMES ​+ ​Ex G2:Ex	G1: 30 G2: 33	G1: 68.4 (7.7) G2: 69.4 (7.7)	NMES	No	6 weeks	6 weeks	VAS pain, WOMAC 10MWT TUG Stair test, maximal quadriceps contraction and voluntary activation
Guidini Lima 2022	Brazil	G1: Laser ​+ ​Ex G2: Ex	G1: 14 G2: 14	G1: 62.0 (9.60) G2: 62.6 (7.5)	Laser	No	6 weeks	3 weeks, 6 weeks and 30 days after end of treatment	VAS Pain, WOMAC, 2 ​min walks test, Sit and Stand test
Gunaydin 2020	Turkey	G1: Shockwave ​+ ​Ex G2: Ex	G1: 18 G2: 20	Mean age 58.8 (6.2)	Shockwave	No	6 weeks	6 and 12 weeks	VAS Pain KOOS 10 ​m walk TUG
Gur 2003	Turkey	G1: Laser @3 ​J ​+ ​Ex G2: Laser @2 ​J ​+ ​Ex G3: Placebo Laser ​+ ​Ex	30 per group	G1: 58.64 (5.92) G2: 59.80 (8.03) G3: 60.52 (6.91)	Laser	Yes	Laser ​= ​2 weeks Exercise ​= ​14 weeks	4, 8, and 12 weeks after last therapy	VAS Pain WOMAC
Hammami 2021	Tunisia	G1: NMES ​+ ​Ex G2: Ex	15 per group	G1: 50.75 (6.92) G2: 50.42 (6.08)	NMES	No	6 weeks	6 weeks	10 ​m walk test sit-to-stand test stair climb test Knee ROM Isokinetic muscle strength KOOS
Imoto 2013	Brazil	G1: NMES ​+ ​Ex G2: Ex	50 per group	G1: 60.60 (6.72) G2: 58.78 (9.60)	NMES	No	8 weeks	8 weeks	NPRS pain Lequense Index TUG
Jorge 2023	Brazil	G1: Laser ​+ ​Ex G2: Placebo laser ​+ ​Ex G2: Ex	G1: 44 G2: 42 G3: 41	G1: 59.1 (9.3) G2: 58.4 (8.3) G3: 59.8 (9.0)	Laser	Yes	8 weeks	8 weeks, 3 months and 6 months	Pain VAS WOMAC 30 ​s chair stand test stair climb test 40 ​m fast paced walk SF-36
Kapci Yildiz 2015	Turkey	G1: Continuous US ​+ ​Ex G2: Pulsed US ​+ ​Ex G3: Placebo US ​+ ​Ex	30 per group	G1: 56.13 (6.61) G2: 54.63 (6.53) G3: 57.76 (7.15)	Continuous US Pulsed US	Yes	8 weeks	8 weeks	VAS pain Lequesne index SF-36
Karadag 2019	Turkey	G1: Heat ​+ ​Ex G2: Ex	15 per group	G1: 57.13 (11.3) G2: 58.73 (10.28)	Heat	No	4 weeks	4 weeks	VAS Pain WOMAC
Karakas 2020	Turkey	G1: Pulsed US ​+ ​Ex G2: Placebo pulsed US ​+ ​Ex	48 per group	G1: 59.1 (7.45) G2: 60.75 (7.46)	US	Yes	8 weeks	8 and 12 weeks	VAS Pain WOMAC TUG Femoral cartilage thickness
Kheshie 2014	Saudi Arabia	G1: High intensity laser ​+ ​Ex G2: Low intensity laser ​+ ​Ex G3: Placebo laser ​+ ​Ex	G1: 20 G2: 18 G3: 15	G1: 52.1 (6.47) G2: 56.56 (7.86) G3: 55.6 (11.02)	High intensity laser Low intensity laser	Yes	6 weeks	6 weeks	VAS Pain WOMAC
Kholvadia 2019	South Africa	G1: Laser ​+ ​Ex G2: Ex	G1: 32 G2: 39	Mean age 61.8 (5)	Laser	No	4 weeks	Post-intervention, 1 and 3 months post-intervention	WOMAC 1-min sit-to-stand Knee ROM
Ones 2006	Turkey	G1: Heat ​+ ​Ex G2: Ex	40 per group	G1: 58.68 (6.79) G2:59.24 (6.92)	Heat	No	15 days	3 weeks and 16 weeks	VAS Pain WOMAC
Park 2021	South Korea	G1: NMES ​+ ​Ex G1: Ex	25 per group	G1: 65.68 (3.24) G2: 66.88 (4.61)	NMES	No	8 weeks	8 weeks	KOOS Isometric muscle strength Biomarkers
Pietrosimone 2011	USA	G1: TENS ​+ ​Ex G2: Placebo TENS ​+ ​Ex G3:Ex	12 per group	NR	TENS	Yes	4 weeks	2 weeks 4 weeks	WOMAC Quadriceps CAR and MVIC
Pietrosimone 2020	USA	G1: TENS ​+ ​Ex G2: Placebo TENS ​+ ​Ex G3: Ex	G1: 32 G2: 29 G3: 29	G1: 60.8 (7.3) G2: 62.5 (7.7) G3: 63 (7.4)	TENS	Yes	4 weeks	2 weeks 4 weeks 8 weeks	WOMAC 20 ​m fast paced walk 30 ​s chair stand Stair climb Quadriceps MVIC and CAR
Quirk 1985	UK	G1: SWD ​+ ​Ex G2: IFT ​+ ​Ex G3: Ex	G1: 12 G2:12 G3:14	G1: 67.2 (SEM 3.2) G2: 59.3 (SEM 3.7)	SWD IFT	No	4 weeks	4 weeks 3 months 6 months	VAS Pain ROM Walking distance Number of stairs
Raeissadat 2018	Iran	G1: EMG biofeedback ​+ ​Ex G2: Ex	23 per group	G1: 60.2 (7.9) G2: 61.9 (9.0)	EMG Biofeedback	No	2 months	2 months	VAS Pain WOMAC VMO thickness on US VMO electrical activity
Rattanachaiyanont 2008	Thailand	G1: SWD ​+ ​Ex G2: Placebo SWD ​+ ​Ex	G1: 53 G2: 60	G1: 63.32 (7.61) G2: 62.48 (8.47)	SWD	Yes	3 weeks	3 weeks 6 weeks	Modified WOMAC 100 ​m walking speed Stair ascent/descent Global Assessment
Sardim 2020	Brazil	G1: Laser ​+ ​Ex G2: Placebo laser ​+ ​Ex	10 per group	G1: 65.1 (1.9) G2: 65.7 (3.2)	Laser	Yes	8 weeks	8 weeks	VAS Pain Lequesne Index SF-36
Stausholm 2023	Norway	G1: Laser ​+ ​Ex G2: Placebo laser ​+ ​Ex	G1: 26 G2: 24	G1: 64.04 (8.52) G2: 61.92 (6.39)	Laser	Yes	8 weeks	3 weeks 8 weeks 26 weeks 52 weeks	VAS Pain KOOS Number of chair stands in 30s Global health change Knee flexion active ROM Knee joint line and tibial condyle pain pressure threshold US of femoral cartilage thickness, suprapatellar effusion, meniscal neovascularization
Vassao 2020 and 2022∗	Brazil	G1: Laser ​+ ​Ex G2: Placebo laser ​+ ​Ex	G1: 17 (∗14) G2: 17 (∗14)	G1: 61.25 (4.34) G2: 61.65 (4.28)	Laser	Yes	8 weeks	8 weeks	NPRS Pain 6 MWT Isometric hip and knee strength ∗WOMAC ∗Inflammatory and cartilage degradation biomarkers
Yilmaz 2010	Turkey	G1: EMG feedback ​+ ​Ex G2: Ex	20 per group	G1: 55.57 (7.17) G2: 59.35 (5.61)	EMG Biofeedback	No	3 weeks	3 weeks	VAS Pain WOMAC Nottingham Health Profile Knee ROM Isokinetic muscle strength
Youssef 2016	Saudi Arabia	G1: Laser 6 ​J/cm^2^+Ex G2: Laser 3 ​J/cm^2^+Ex G3: Placebo Laser ​+ ​Ex	20 per group	G1: 67.3 (2.9) G2: 67.5 (2.5)	Laser (at 2 different doses)	Yes	8 weeks	8 weeks	VAS Pain WOMAC Knee ROM Isometric muscle strength

6MWT, Six-minute walk test; CAR, Central Activation Ratio; EMG, Electromyographic; Ex. Exercise G1, Group 1; G2, Group 2; GMed, Gluteus Medius; HILT, High Intensity Laser Therapy; IFT, Interferential Therapy; J, Joules; KOOS, Knee Osteoarthritis Outcome Score; MVIC, Maximum Voluntary Isometric Contraction; NMES, Neuromuscular Electrical Stimulation; NPRS, Numerical Pain Rating Scale; NR, Not Reported; PEME, Pulsed Electromagnetic Energy; ROM, Range of Motion; SF-36, Short-Form 36; TENS, Transcutaneous Electrical Nerve Stimulation; TUG, Timed Up and Go; US, Ultrasound; VAS, Visual Analogue Scale; VMO, Vastus Medius Oblique; WOMAC, Western Ontario and McMaster Universities Osteoarthritis Index.

### 3.3 Interventions

Nine EPTs were investigated, including TENS [34,35], laser therapy [10,[22], [23], [24],^28^,^30^,^31^,^38^,^41^,^42^,^51^,^62^], IFT [9,25,47,52], US [36,52,55], shortwave [26,45,47,52], shockwave [46,59], heat therapy [44,50,57], NMES [20,63] and EMG biofeedback [48,49]. Interventions varied between 8 and 60 treatments, delivered between 2 weeks and 11 months. No trials reported intervention content in line with the Template for Intervention Description and Replication (TIDieR) guidelines, developed in 2014 to improve the reporting of interventions [64], despite 20 (50%) trials published after 2014. Intervention details are provided in Supplemental file 3. Details on what, how, where and by whom interventions were delivered varied across studies. Just three studies (8%) reported how treatment adherence/fidelity would be measured, whilst actual intervention adherence/fidelity was reported by 14/40 studies (35%).

### 3.4 Meta-analysis

As data could not be retrieved from one study [47], 39 studies were included in the meta-analysis. All studies reported end-of-treatment scores or provided relevant data on request [33], except for one, which reported change scores only [52].

### 3.5 Adjunctive EPTs and exercise therapy vs placebo EPTs and exercise therapy pain

**Fig. 2:**
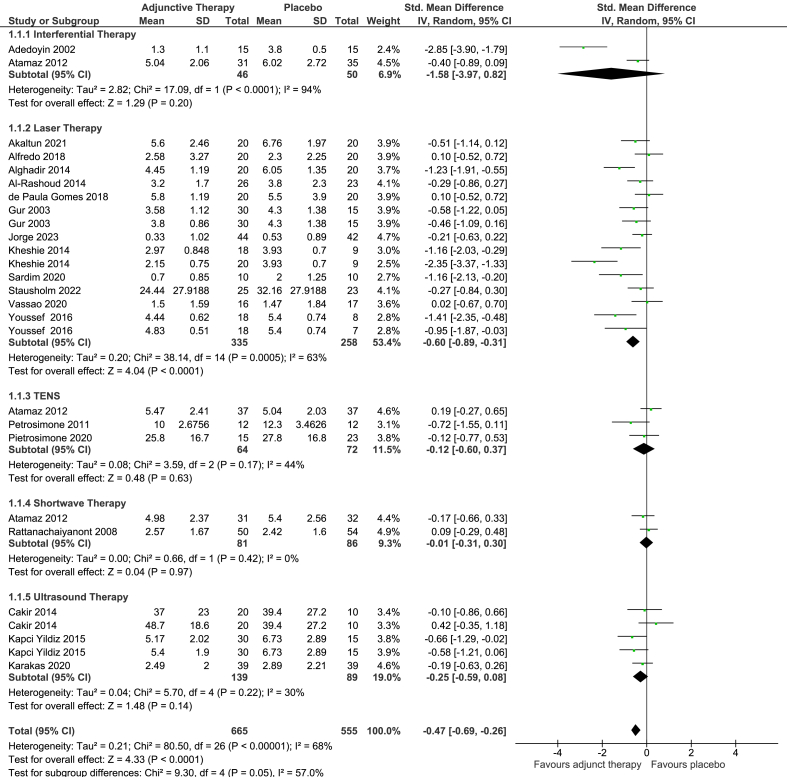
Forest plot of EPT plus Exercise therapy versus Placebo EPT plus Exercise therapy: pain outcome (short-term).

The overall effect estimate across all EPTs for pain post-intervention (short-term) (SMD -0.47, 95% CI -0.69 to −0.26; NPRS MD -0.87, 95% CI -1.28 to −0.48) in favour of EPTs plus exercise (20 trials, 26 comparisons), favouring EPT and exercise therapy was statistically significant (Fig. 2). Heterogeneity was substantial (I^2^ ​= ​68%). The relative change was −11% (95% CI -16% to −7%). These results are clinically unimportant as the 95% CIs do not include the MCIDs of 2 points/15%. Across subgroups, only laser therapy plus exercise showed a statistically significant effect (SMD -0.60, 95% CI -0.89 to −0.31; NPRS MD -1.04, 95% −1.54 to −0.54) compared to the placebo comparison in 12 trials (15 comparisons). The relative change was −15% (95% CI -23% to −8%). Heterogeneity was substantial (I^2^ ​= ​63%). Inclusion of the backtranslated mean effect, upper 95% CI and proximity of the lower 95% CI to the MCID of 6 points or 15% indicates that this finding could be clinically important. There were no significant differences between IFT, US, TENS or shortwave used in combination with exercise compared to their placebo counterpart and exercise (Fig. 2).

Five studies (nine comparisons) evaluated medium-term effects on pain. Overall pooling of findings resulted in a SMD of −0.22 (95% CI -0.45 to 0.01) in favour of EPTs, which was not statistically significant. Heterogeneity was moderate (I^2^ ​= ​33%). Subgroup analysis of three different EPTs; laser (four trials, SMD -0.37, 95% CI -0.77 to 0.04), shortwave (one trial, SMD 0.08, 95% CI -0.41 to 0.58), IFT (one trial, SMD -0.51, 95% CI -1.01 to −0.02), TENS (one trial, SMD 0.01, 95% CI -0.45 to 0.46) and US (one trial, two comparisons, SMD 0.00, 95% CI -0.53 to 0.54) also demonstrated no between-group differences (Supplemental file 4).

One trial evaluated long-term effects of laser therapy, with no statistically significant difference between the groups based on a 0–100 VAS (MD 4.88, 95% CI -11.07 to 20.83) (Supplemental file 4).

### 3.6 Physical function

**Fig. 3:**
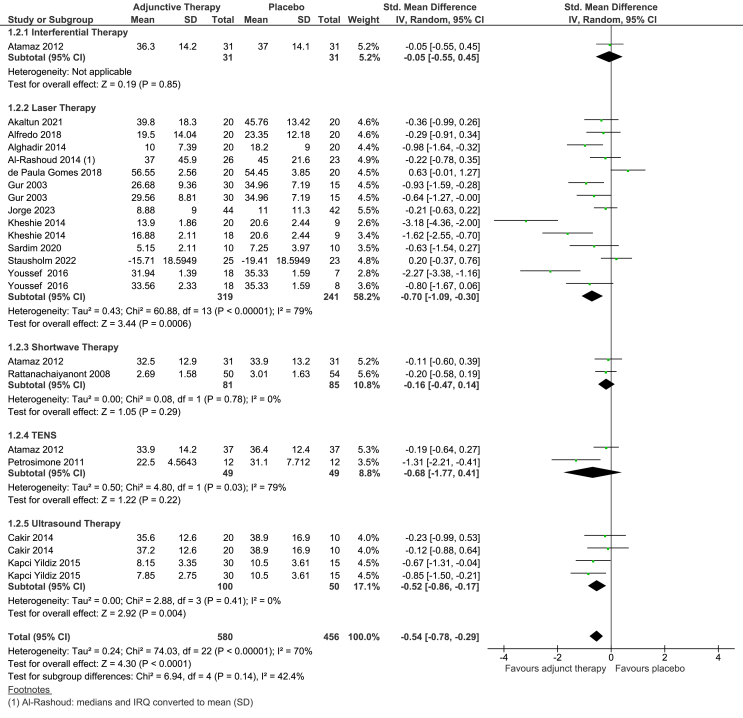
Forest plot of EPT plus Exercise therapy versus Placebo EPT plus Exercise therapy: physical outcome (short-term).

The overall effect estimate (SMD -0.54, 95% CI -0.78 to −0.29; WOMAC MD -6.32, 95% CI -9.13 to −3.39) was statistically significant in favour of EPTs plus exercise compared to placebo EPTs and exercise in the short-term (Fig. 3). The relative change was −15% (95% CI -21% to −8%). Due to inclusion of the backtranslated mean effect, upper 95% CI and proximity of the lower 95% CI in the MCID threshold, this finding could be clinically important. Heterogeneity was substantial (I^2^ ​=70%).

There was a statistically significant effect in favour of laser therapy compared to placebo (SMD -0.70, 95% CI -1.09 to −0.30; WOMAC MD -10.79, 95% CI -16.81 to −4.63) based on 11 trials (14 comparisons). The relative change was −21% (95% CI -33% to −9%). Heterogeneity was considerable (I^2^ ​= ​79%). Due to inclusion of the backtranslated mean effect, upper 95% CI and proximity of the lower 95% CI in the MCID of 6 points/15%, this finding could be clinically important. Both pain and physical function results were influenced by one trial which reported SMDs >2 [38] (Fig. 2, Fig. 3).

There was a statistically significant effect in favour of US plus exercise compared to placebo US plus exercise (SMD -0.52, 95% CI -0.86 to −0.17; WOMAC MD -8.01, 95% CI -13.24 to −2.62), based on two trials (four comparisons), with negligible heterogeneity (I^2^ ​= ​0). Relative change was −15% (95% CI -25% to −5%). Results are clinically unimportant as the 95% CIs do not include the MCID (6points/15%). There were no significant differences between IFT, TENS or shortwave therapy combined with exercise compared to placebo EPT/exercise (Fig. 3).

Six studies (nine comparisons) evaluated medium-term effects on physical function. Overall pooled estimate (SMD -0.13, 95% CI -0.31 to 0.05) was not statistically significant, with negligible heterogeneity (I^2^ ​= ​0%). Pooled results of subgroups of five different EPTs showed no statistically significant difference between-group differences (Supplemental file 5).

One trial evaluated long-term effects of laser therapy, with no statistically significant difference between the groups based on a 0–100 KOOS disability subscale (MD 6.28, 95% CI -6.35 to 18.91). (Supplemental file 5).

### 3.7 Quality of Life

Three studies evaluated short-term effects on QoL [41]. Overall pooled estimate (SMD 0.30, 95% CI -0.02 to 0.62) was not statistically significant. Heterogeneity was negligible (I^2^ ​= ​0) (Supplemental file 6).

Two studies of laser therapy evaluated medium-term effects on QoL resulting in a non-signficant pooled SMD of −0.10 (95% CI -0.44 to 0.24), with negligible heterogeneity (I^2^ ​= ​0%) (Supplemental file 6).

One trial evaluated long-term effects, with no statistically significant between-group differences based on a 0–100 KOOS QoL subscale (MD 6.59, 95% CI -4.96 to 18.14) (Supplemental file 6).

### 3.8 Global rating of change

One trial evaluated global assessment post-intervention [23]. A total of 81% improved in the laser therapy group compared to 87% in the placebo group, resulting in a non-significant risk ratio (RR) of 0.92 (95% CI 0.73 to 1.17) (Supplemental file 7).

### 3.9 Adverse events

One study reported on adverse events [33], with an overall RR of 1.18 [95% CI 0.76 to 1.81] (Supplemental file 8). Shortwave-related events comprised mild pain (n ​= ​3), and worsening pain requiring surgery (n ​= ​1) in the intervention group, and mild pain (n ​= ​3), mild swelling (n ​= ​1) and feeling of vasodilatation (n ​= ​1) in the placebo SWD group. Exercise-related adverse events in the intervention group were increased crepitus (n ​= ​8), mild muscle tightness (n ​= ​16), compared with increased crepitus (n ​= ​6), mild muscle tightness (n ​= ​13), fatigue (n ​= ​3) and mild pain (n ​= ​4) in the placebo group.

### 3.10 Adjunctive EPTs plus exercise therapy versus exercise therapy only pain

**Fig. 4:**
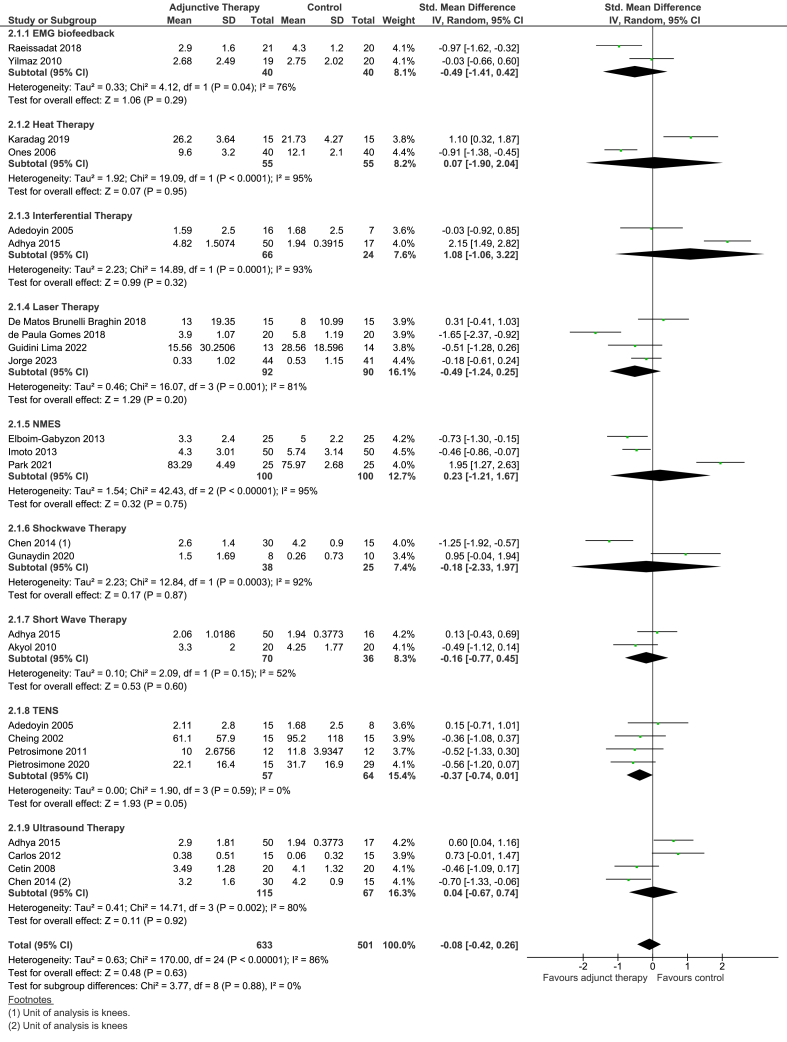
Forest plot of EPT plus Exercise therapy versus Exercise therapy: pain outcome (short-term).

For short-term effects, the overall effect estimate of SMD -0.08 (95% CI -0.42 to 0.26) was not statistically significant, based on 21 trials (25 comparisons). Heterogeneity was considerable (I^2^ ​= ​86%). No subgroups (laser, US, TENS, shortwave, shockwave, NMES, heat and EMG biofeedback) showed a statistically significant difference between the two groups (Fig. 4).

One trial evaluated medium-term effects of laser therapy, with no statistically significant difference between the groups based on a 0–10 VAS (MD -0.50, 95% CI -1.28 to 0.28) (Supplemental file 9).

### 3.11 Physical function

The overall effect estimate for physical function was a SMD of −0.17 (95% CI -0.43 to 0.09, based on 21 trials (26 comparisons). Heterogeneity was considerable (I^2^ ​= ​78%), which was not statistically significant.

**Fig. 5:**
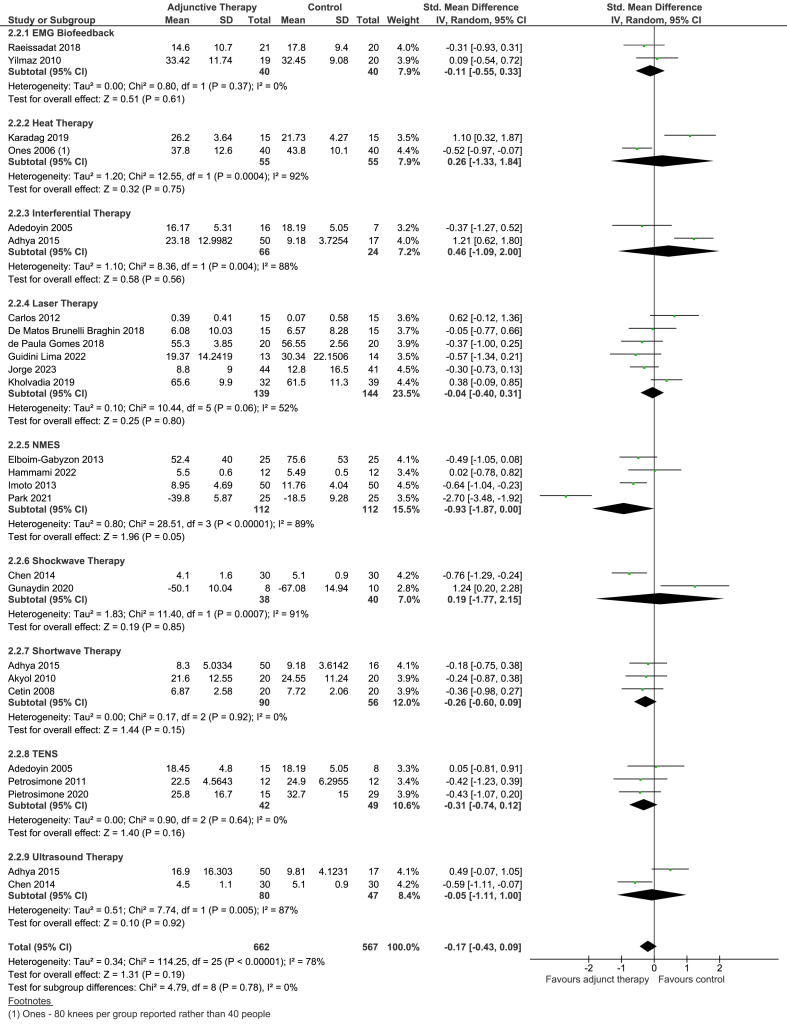
Forest plot of EPT plus Exercise therapy versus Exercise therapy: physical function outcome (short-term).

None of the subgroups of EMG biofeedback, heat therapy, laser, IFT, shockwave or shortwave showed statistically significant differences between the two groups (Fig. 5).

Two trials evaluated medium-term effects of laser therapy, with no statistically significant difference between the groups (SMD 0.23, 95% CI -0.68 to 1.15) (Supplemental file 9).

### 3.12 Quality of Life

Three trials evaluated short-term effects on QoL, resulting in a SMD of −0.67 (95% CI -1.16 to −0.18) in favour of adjunctive therapy, with moderate heterogeneity (I^2^ ​= ​51%). When backtranslated to a 0–100 SF-36 mental health scale, the mean difference was −6.22 (95% CI -10.76 to −1.67) and a relative change of −40% (95% CI -89% to −9%), which was clinically unimportant based on MCID threshold values.

Two trials, which evaluated NMES [19,20] found no statistically significant difference in QoL between adjunctive NEMS plus exercise versus exercise (SMD -0.76, 95% CI -1.64 to 0.11). Heterogeneity was substantial (I^2^ ​= ​67%) (Supplemental file 9).

One trial resulted in a statistically significant effect favouring adjunctive laser therapy (SMD -0.51 (95% CI -0.94 to −0.07; SF-36 MD -5.80, 95% CI -10.73 to −0.87) and a relative difference of −9% (95% CI -17% to −1%), which was not clinically significant (Supplemental file 9).

One trial of laser therapy evaluated medium-term effects on QoL, resulting in a MD -6.20 (95% CI -14.04 to 1.64), based on a 0–100 SF-36 mental health scale, which was not statistically significant (Supplemental file 9).

### 3.13 Adverse events

No trials in the EPT and exercise versus exercise only comparison reported on adverse events or withdrawals due to adverse effects.

### 3.14 Other secondary outcomes

Radiographic joint structure changes were not reported in any of the 40 trials.

## 4 Risk of bias and certainty of evidence

**Fig. 6:**
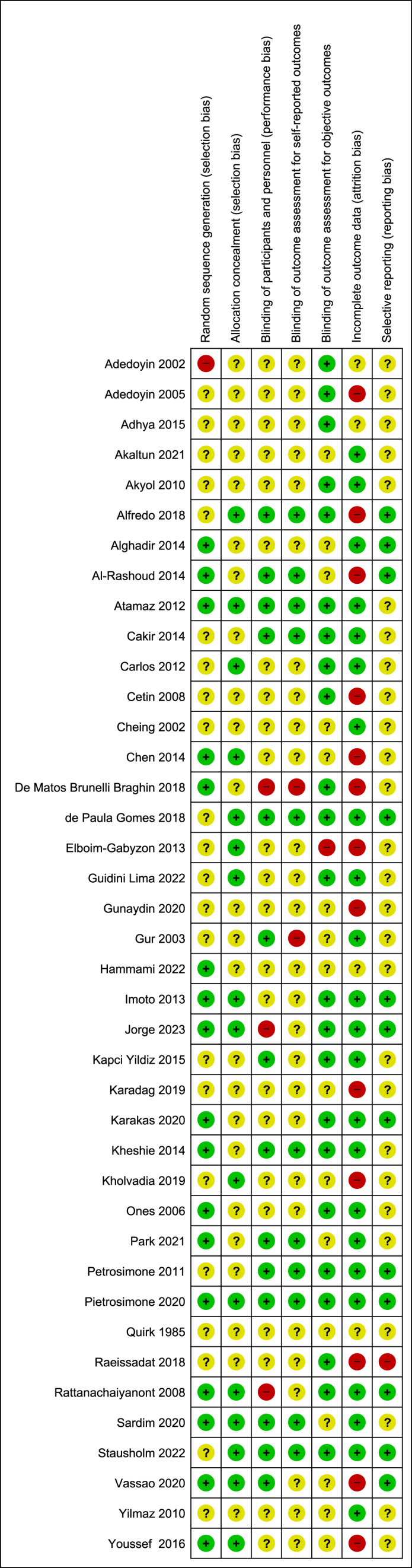
Risk of Bias assessment.

A summary of RoB judgements across domains associated with selection performance, detection attrition and reporting bias is shown in Fig. 6. For selection bias, 43% (17/40) trials were rated as low risk, based on random sequence generation, and 40% (16/40) were low risk based on allocation concealment. Just 35% (14/40) were deemed to be at low risk of performance bias, based on blinding of participants and personnel. Detection bias was assessed based on reporting of self-reported outcomes, with 30% (12/40) at low risk of bias, and 60% (24/40) rated as low risk of detection bias based on assessment of objective outcomes. A total of 60% (24/40) was rated as low risk of attrition bias, and 30% (12/40) were at low risk of reporting bias.

Using the GRADE approach to assess certainty of evidence for all EPTs combined and EPT subgroups, certainty of evidence ranged from ‘moderate’ to ‘very low’. Publication bias could only be assessed for all EPTs and laser therapy, as 10 or more trials were included. Details of the GRADE assessment are available in Table 2 and funnel plots are in Supplemental file 10.

**Table 2:** GRADE Summary of Findings tables.

(a) Adjunctive Electrophysical Therapy and Exercise Therapy vs. Placebo Adjunctive Electrophysical Therapy and Exercise Therapy								
	Time-frame and Outcome	Number of studies	Risk of bias	Inconsistency	Indirectness	Imprecision	Publication Bias	Overall Certainty
All EPTs	Short-term Pain	20	Serious	Serious	Not serious	Not serious	Strongly suspected	Low
Interferential Therapy	Short-term Pain	2	Serious	Serious	Not serious	Serious	Not assessed	Very low
Laser Therapy	Short-term Pain	12	Serious	Serious	Not serious	Not serious	Strongly suspected	Very low
Shortwave	Short-term Pain	1	Not serious	Not serious	Not serious	Serious	Not assessed	Moderate
TENS	Short-term Pain	3	Not serious	Not serious	Not serious	Serious	Not assessed	Moderate
Ultrasound	Short-term Pain	3	Serious	Not serious	Not serious	Serious	Not assessed	Low
All EPTs	Short-term –Physical Function	17	Serious	Serious	Not serious	Serious	Strongly suspected	Very low
Interferential Therapy	Short-term –Physical Function	1	Not serious	Not assessed	Not serious	Serious	Not assessed	Low
Laser Therapy	Short-term –Physical Function	12	Serious	Very Serious	Not serious	Serious	Not assessed	Very low
Shortwave	Short-term –Physical Function	2	Serious	Serious	Not serious	Serious	Not assessed	Very low
TENS	Short-term –Physical Function	2	Serious	Serious	Not serious	Serious	Not assessed	Very low
Ultrasound	Short-term –Physical Function	2	Serious	Not serious	Not serious	Serious	Not assessed	Low
All EPTs/laser	Short-term QOL	3	Serious	Not serious	Not serious	Serious	Not assessed	Low
All EPTs	Medium-term -Pain	6	Serious	Not serious	Not serious	Serious	Not assessed	Low
Interferential Therapy	Medium-term -Pain	1	Not serious	Not assessed	Not serious	Serious	Not assessed	Low
Laser	Medium-term -Pain	4	Serious	Serious	Not serious	Serious	Not assessed	Very Low
Shortwave	Medium-term -Pain	1	Not serious	Not assessed	Not serious	Serious	Not assessed	Low
TENS	Medium-term -Pain	1	Not serious	Not assessed	Not serious	Serious	Not assessed	Low
US	Medium-term -Pain	1	Serious	Not assessed	Not serious	Serious	Not assessed	Very low
All EPTs	Medium-term –Physical Function	6	Serious	Not serious	Not serious	Serious	Not assessed	Low
Interferential Therapy	Medium-term –Physical Function	1	Not serious	Not assessed	Not serious	Serious	Not assessed	Low
Laser	Medium-term –Physical Function	4	Not serious	Not serious	Not serious	Serious	Not assessed	Low
Shortwave	Medium-term –Physical Function	1	Not serious	Not assessed	Not serious	Serious	Not assessed	Low
TENS	Medium-term –Physical Function	1	Not serious	Not assessed	Not serious	Serious	Not assessed	Low
Ultrasound	Medium-term –Physical Function	1	Serious	Not serious	Not serious	Serious	Not assessed	Low
All EPTs/laser	Medium-term QOL	2	Serious	Not serious	Not serious	Serious	Not assessed	Low
Laser Therapy	Long-term - Pain	1	Not serious	Not assessed	Not serious	Serious	Not assessed	Low
	Long-term Physical Function	1	Not serious	Not assessed	Not serious	Serious	Not assessed	Low
	Long-term Quality of Life	1	Not serious	Not assessed	Not serious	Serious	Not assessed	Low

(b) Adjunctive Electrophysical Therapy and Exercise Therapy vs. Exercise Therapy								
		Number of studies	Risk of bias	Inconsistency	Indirectness	Imprecision	Publication Bias	Overall Certainty
All EPTs	Short-term -Pain	21	Serious	Serious	Not serious	Serious	Strongly suspected	Very low
EMG biofeedback	Short-term -Pain	1	Serious	Serious	Not serious	Serious	Not assessed	Very low
Heat Therapy	Short-term -Pain	2	Serious	Very serious	Not serious	Very serious	Not assessed	Very low
Interferential Therapy	Short-term -Pain	2	Serious	Very serious	Not serious	Very serious	Not assessed	Very low
Laser Therapy	Short-term -Pain	4	Serious	Serious	Not serious	Serious	Not assessed	Very low
NMES	Short-term -Pain	3	Serious	Very serious	Not serious	Serious	Not assessed	Very low
Shockwave	Short-term -Pain	2	Serious	Very serious	Not serious	Very serious	Not assessed	Very low
Shortwave	Short-term -Pain	2	Serious	Serious	Not Serious	Serious	Not assessed	Very low
TENS	Short-term -Pain	3	Serious	Not serious	Not Serious	Serious	Not assessed	Low
Ultrasound	Short-term -Pain	3	Serious	Serious	Not serious	Serious	Not assessed	Very low
All EPTs	Short-term –Physical Function	17	Not serious	Serious	Not serious	Serious	Strongly suspected	Very low
EMG biofeedback	Short-term –Physical Function	2	Serious	Not serious	Not serious	Serious	Not assessed	Low
Heat Therapy	Short-term –Physical Function	2	Serious	Very serious	Not serious	Very serious	Not assessed	Very low
Interferential Therapy	Short-term –Physical Function	2	Serious	Very serious	Not serious	Very serious	Not assessed	Very low
Laser Therapy	Short-term –Physical Function	7	Serious	Serious	Not serious	Serious	Not assessed	Very low
NMES	Short-term –Physical Function	4	Serious	Very serious	Not serious	Serious	Not assessed	Very low
Shockwave	Short-term –Physical Function	2	Serious	Very serious	Not serious	Very serious	Not assessed	Very low
Shortwave	Short-term –Physical Function	3	Serious	Not serious	Not serious	Serious	Not assessed	Low
TENS	Short-term –Physical Function	3	Serious	Not serious	Not Serious	Serious	Not assessed	Low
Ultrasound	Short-term –Physical Function	2	Serious	Serious	Not serious	Serious	Not assessed	Low
All EPTs	Short-term QOL	3	Serious	Serious	Not serious	Serious	Not assessed	Very low
NMES	Short-term QOL	2	Serious	Serious	Not serious	Serious	Not assessed	Very low
Laser	Short-term QOL	1	Serious	Not assessed	Not serious	Serious	Not assessed	Very low
All EPTs/Laser	Medium-term Pain	1	Serious	Not assessed	Not serious	Serious	Not assessed	Very low
All EPTs/Laser	Medium-term Physical Function	2	Serious	Serious	Not serious	Serious	Not assessed	Very low
All EPTs/Laser	Medium-term QOL	1	Serious	Not assessed	Not serious	Serious	Not assessed	Very low

EMG, Electromyographic; EPTs, Electrophysical Therapies; IFT, Interferential Therapy; NMES, Neuromuscular Electrical Stimulation; TENS; Transcutaneous Electrical Nerve Stimulation; US, Ultrasound.

GRADE Working Group grades of evidence (16)

• **High certainty:** we are very confident that the true effect lies close to that of the estimate of the effect.

• **Moderate certainty:** we are moderately confident in the effect estimate: the true effect is likely to be close to the estimate of the effect, but there is a possibility that it is substantially different.

• **Low certainty:** our confidence in the effect estimate is limited: the true effect may be substantially different from the estimate of the effect.

• **Very low certainty:** we have very little confidence in the effect estimate: the true effect is likely to be substantially different from the estimate of effect.

## 5 Discussion

This systematic review synthesised the evidence for different EPTs used adjunctively with exercise therapy for hip or knee OA. No trials investigated hip OA, so findings relate only to knee OA. Our original Cochrane review found, in subgroup analysis, statistically significant, but clinically unimportant, differences between adjunctive EPTs and corresponding placebo therapies in pain and function outcomes in the short-term [5] and non-significant differences in QoL. Five additional trials added for this review [11,19,20,23,65] did not change results for overall effects.

However, this analysis aimed to primarily elucidate the effectiveness of different types of EPTs. Based on our first comparison against the equivalent placebo therapy used with exercise therapy, only adjunctive laser therapy showed a statistically significant benefit in pain and physical function, which may be clinically important and based on very-low certainty of evidence, using the GRADE criteria. Therefore, these results should be cautiously interpreted.

For the second comparison of adjunctive EPTs and exercise therapy versus exercise therapy only, we found no statistically significant overall or subgroup effect across any EPTs.

Comparison with findings from other systematic reviews of EPTs, used adjunctively with exercise is limited, as their comparator intervention could have included a no-treatment control, or other non-exercise interventions [[66], [67], [68], [69], [70]]. One review of NMES, based on six included trials, concluded that NEMS added to exercise demonstrated moderate evidence for improvements in muscle strength, but pain, function or QoL were not evaluated [71]. Two recent reviews, which compared laser therapy as an adjunct to exercise, against exercise, both reported significant improvements in pain favouring laser therapy [72,73]. They deemed these findings as clinically significant based on a mean difference (MD) of 13.41 (95% CI 5.46 to 21.37) on a VAS 0–100 pain scale [72], and a SMD of −0.55 (95% CI -0.88 to −0.22) [73]. Back-translating SMDs, as we did in this review, increases the interpretability of pooled effect estimates into a more clinically meaningful result, but can be problematic as it potentially dichotomises treatments into ‘worthwhile/not worthwhile’. A more nuanced approach that considers the complexity of the clinical encounter and patient wishes is suggested [74]. Various MCIDs for pain severity have been used in knee OA reviews and guidelines, ranging from 10 to 19 on a VAS scale, resulting in conflicting treatment recommendations, rejecting treatments that may be effective, or accepting ineffective treatments [75].

At a physiological level, laser therapy is purported to modulate inflammatory processes and increase cellular proliferation [76,77] to improve pain and physical function in hip or knee OA.

In addition to physiological effects, which may translate into therapeutic effects, contextual factors, as potential contributors to treatment effects can include placebo effects, natural history and co-therapies, and have been shown to contribute a proportion of analgesic effects in non-pharmacological knee OA trials [78]. Our inclusion only of studies with identical exercise in treatment and control groups may limit the role of contextual factors, although other factors, including patients' expectation, clinicians’ behaviour and interaction [79], as contributors to contextual effects, cannot be accounted for in these trials.

Whilst evidence demonstrates the physiological effects of EPTs on body tissues [80], various reasons for lack of translation into clinical effects in trials include insufficient dosage [80], methodological issues including outcomes assessed, timing of outcome measurement and comparison groups used [81]. The low quality of evidence of studies in this review is of concern, associated with the high proportion of trials demonstrating high or unclear risk of selection, performance, detection or attrition bias. The low sample sizes is also concerning.

Poor reporting of adverse events is also noteworthy, along with ambiguity around the nature of the events reported i.e., serious/non-serious, related/unrelated to the interventions, and whether there were resultant trial withdrawals. This is consistent with a systematic review, which identified inadequacies including adverse events assessment, reporting of withdrawals and details of adverse events in non-pharmacological trials [82].

## 6 Review strengths and limitations

Strengths include a comprehensive search strategy using six bibliographic databases, without English language restrictions. Risk of bias assessment and certainty of evidence using the Cochrane RoB and GRADE tools, was undertaken by two independent review authors. We included only studies where the exercise was identical in both control and intervention arms, thereby evaluating the evidence for the additive effect of EPTs to exercise therapy.

The high heterogeneity and low certainty of evidence is a limitation, along with variation in clinical parameters used, within EPT subgroups, creating clinical heterogeneity. Most trials assessed short-term outcomes only, with only six trials assessing medium-term (<6 months) effects, and one assessing long-term (>6 months) effects.

## 7 Implications for research and practice

No trials investigated hip OA, so results only apply to individuals with knee OA. Findings suggest a potential role for laser therapy alongside exercise therapy for knee OA for improving pain and physical function, however, results relate to short-term effects. Future RCTs should enhance methodological rigour by addressing various sources of bias. Robust, realistic sample size estimations should be undertaken a priori [83] and intervention fidelity should be evaluated. Trialists should review OARSI recommendations when designing and undertaking future clinical trials of EPTs [84]. With recognition of OA as a chronic condition, longer-term follow-ups beyond six months are essential to provide meaningful results. Sufficient dosage to provide an optimal treatment effect should be a key consideration in future EPT trials. For example, greater treatment effects have been reported for knee OA trials of laser dosages aligned with the World Association of Laser Therapy (WALT) dosage recommendations [72]. Future trials should follow the TIDieR [64] checklist to improve reporting of interventions.

## 8 Conclusion

Results suggest that across various EPTs used adjunctively with exercise therapy for knee OA, adjunctive laser therapy probably confers a clinical benefit in pain and physical function, when compared to placebo adjunctive laser, based on very-low certainty evidence. None of the other EPTs evaluated demonstrated clinically meaningful effects in pain and function over and above exercise therapy used either with a placebo EPT or on its own. Certainty of evidence was most commonly low or very low, and rarely moderate, across different comparisons. Results are predominantly based on short-term effects with few trials evaluating effects beyond six months.

## Author contributions

Concept: HPF.

Methodology: HPF, JC, RG, SA.

Formal analysis: HPF, JC, RG, SA.

Writing- original draft preparation: HPF.

Writing-reviewing and editing: HPF, JC, RG, SA.

HPF takes responsibility for the integrity of the work as whole.

## Conflict of interest

The authors confirm they have no conflict of interest.

## Funding

This research received no external funding.

## Acknowledgements

We would like to thank Tamara Radar, Information Specialist for the Cochrane Musculoskeletal group, for designing the search strategy.
