# Supplementary material for: Adjunctive electrophysical therapies used in addition to land-based exercise therapy for osteoarthritis of the hip or knee: A systematic review and meta-analysis
Source: Osteoarthr Cartil Open. 2024 Mar 1;6(2):100457. doi: 10.1016/j.ocarto.2024.100457 (PMC10956074; doi:10.1016/j.ocarto.2024.100457)
Supplement: Multimedia component 1 [file mmc1.docx]

**List of Supplementary Files**

1. Supplemental File 1: Medline search strategy
2. Supplemental file 2: GRADE Assessment Criteria
3. Supplemental File 3: TIDIER checklist
4. Supplemental File 4: Forest Plots- EPT plus Exercise therapy versus Placebo EPT plus Exercise therapy (Pain outcome)
5. Supplemental File 5: Forest Plots- EPT plus Exercise therapy versus Placebo EPT plus Exercise therapy (Physical Function outcome)
6. Supplemental File 7: Forest Plots- EPT plus Exercise therapy versus Placebo EPT plus Exercise therapy (Global Rating of Change outcome)
7. Supplemental File 8: Forest Plots- EPT plus Exercise therapy versus Placebo EPT plus Exercise therapy (Adverse Events)
8. Supplemental File 9: Forest Plots- EPT plus Exercise therapy versus Exercise therapy (Pain and Physical Function and Quality of Life)
9. Supplemental File 10: Funnel Plots
