# Supplementary material for: Adjunctive electrophysical therapies used in addition to land-based exercise therapy for osteoarthritis of the hip or knee: A systematic review and meta-analysis
Source: Osteoarthr Cartil Open. 2024 Mar 1;6(2):100457. doi: 10.1016/j.ocarto.2024.100457 (PMC10956074; doi:10.1016/j.ocarto.2024.100457)
Supplement: Multimedia component 2 [file mmc2.docx]

**Supplemental File 1: Medline search strategy**

1 exp osteoarthritis/

2 osteoarthr$.tw.

3 (degenerative adj2 arthritis).tw.

4 arthrosis.tw.

5 or/1-4

6 knee/

7 exp knee Joint/

8 knee$.tw.

9 hip/

10 exp hip joint/

11 hip$.tw.

12 or/6-11

13 5 and 12

14 exp EXERCISE/

15 exp exertions/

16 exp Physical Fitness/

17 exp Exercise Test/

18 exp Exercise Tolerance/

19 exp sports/

20 exp PLIABILITY/

21 exp Physical Endurance/

22 exertion$.tw.

23 exercis$.tw.

24 sport$.tw.

25 ((physical or motion) adj5 (fitness or therap$)).tw.

26 (physical$ adj2 endur$).tw.

27 ((strength$ or isometric$ or isotonic$ or isokinetic$ or aerobic$ or endurance or weight$) adj5 (exercis$ or train$)).tw.

28 exp physical therapy modalities/

29 physiotherap$.tw.

30 manipulat$.tw.

31 kinesiotherap$.tw.

32 exp Rehabilitation/

33 rehab$.tw.

34 (skate$ or skating).tw.

35 run$.tw.

36 jog$.tw.

37 treadmill$.tw.

38 swim$.tw.

39 bicycl$.tw.

40 (cycle$ or cycling).tw.

41 walk$.tw.

42 (row or rows or rowing).tw.

43 muscle strength$.tw.

44 or/14-43

45 13 and 44

46 randomized controlled trial.pt.

47 controlled clinical trial.pt.

48 randomized.ab.

49 placebo.ab.

50 clinical trials as topic.sh.

51 randomly.ab.

52 trial.ti.

53 or/46-52

54 exp animals/ not humans.sh.

55 53 not 54

56 45 and 55
