# Supplementary material for: Adjunctive electrophysical therapies used in addition to land-based exercise therapy for osteoarthritis of the hip or knee: A systematic review and meta-analysis
Source: Osteoarthr Cartil Open. 2024 Mar 1;6(2):100457. doi: 10.1016/j.ocarto.2024.100457 (PMC10956074; doi:10.1016/j.ocarto.2024.100457)
Supplement: Multimedia component 3 [file mmc3.docx]

**Supplemental file 2: GRADE Assessment Criteria**

- Risk of Bias was assessed by inspection of the risk of bias summary across trials. Risk of bias was considered serious if evidence was mostly from studies of unclear/high risk of from one domain (i.e., selection, performance, detection, attrition or reporting), and very serious if evidence was mostly from studies of unclear/high risk of bias on multiple categories
- Inconsistency: was determined by assessment of the 95% CIs of the individual studies, whereby studies were downgraded if there was wide variance in the point estimates across studies, minimal or no overlap of the 95% confidence intervals or high levels of heterogeneity indicated by the I^2^ statistic. We also downgraded if inconsistency could not be assessed if there was only one study.
- Indirectness: We downgraded studies if a surrogate measure of outcome was used, or if the comparison between treatment and control/placebo was not head-to-head.
- Imprecision: We downgraded studies were downgraded if the pooled sample size was <400 or if the pooled 95% CI crossed the line of no effect, or the width of the 95% CI exceeded a standardised mean difference of 0.5
- Publication bias was assessed only if there were 10 or more trials, and was downgraded if the funnel plot demonstrated asymmetry.
