# Supplementary material for: Adjunctive electrophysical therapies used in addition to land-based exercise therapy for osteoarthritis of the hip or knee: A systematic review and meta-analysis
Source: Osteoarthr Cartil Open. 2024 Mar 1;6(2):100457. doi: 10.1016/j.ocarto.2024.100457 (PMC10956074; doi:10.1016/j.ocarto.2024.100457)
Supplement: Multimedia component 4 [file mmc4.docx]

**Supplemental File 3: TIDIER checklist**

| **Author year** | **WHY** | **WHAT/ HOW** | **WHO provided** | **WHERE** | **WHEN AND HOW MUCH** | **TAILORING** | **MODIFICATIONS** | **HOW WELL planned** | **HOW WELL Actual** |
| --- | --- | --- | --- | --- | --- | --- | --- | --- | --- |
| Adedoyin 2002 | Pain relief | **IFT:** Frequency of 100Hz, pulse length of 1/30 of 1 second for the first 15 minutes of treatment. Intensity of the stimulus was gradually increased until patients felt an appreciable sensation. Stimulus was reduced to 80 Hz for the next 5 minutes. | Physiotherapist | Physiotherapy department | 8 x 20-minute treatments over 4 weeks | NR | NR | NR | NR |
| Adedoyin 2005 | Pain relief | **IFT:** Two electrodes, with moist pads, were applied to either side of the knee and aligned longitudinally with the limb and secured with Velcro straps. Beat frequency of 80 Hz. The intensity of the stimulus was gradually increased until the patients felt a strong but comfortable sensation.  **TENS:** procedure and application of TENS was precisely the same as for IFT. Stimulation frequency was  80 Hz continuous, phase duration was 200 ms. Current intensity was strong but comfortable. A different  therapist used IFT and TENS for all patients. | Physiotherapist | Physiotherapy department | 8 x 20-minute treatments over 4 weeks with at least 24 hours between each session | NR | NR | NR | NR |
| Adhya 2015 | Relieve pain and improve function | **PEME:** Frequency of 27.12 MHz, 11 m waves of 150 watts for 15 minutes.  **US:** treatment over knee tender points based on clinical examination. Frequency of 1.1 MHz, duty cycle 100%, effective radiating area (ERA) 4.0 cm^2^, intensity 1.00 W/cm^2^, treatment time 7.30 minutes.  **IFT:** Frequency of 100 Hz, pulse length of 1/30^th^ of 1 second for the first 15 minutes. Stimulus was then reduced to 80 Hz for the next 5 minutes. Intensity of the stimulus was gradually increased until the patient felt moderate pins and needles. | NR | Physiotherapy department | 3 times a week for 8 weeks (total 24 treatments) | NR | NR | NR | NR |
| Akaltun 2021 | Improve clinical outcomes | **Laser therapy:** 1064nm wavelength high intensity laser, with a maximum power of 12 W. Biostimulation and analgesic modes were delivered using continuous circular motions. The analgesic mode was applied for treatments 1 to 3. A total of 300 J was applied as 12 J/m^2^ , 25 cm^2^ at a frequency of 25 Hz. The biostimulation mode was used for treatments 4 to 10, with total of 3000 J applied as 120 J/cm^2^ in this mode. | NR | NR | 5 treatments per week for 2 weeks (total of 10 treatments). | NR | NR | NR | NR |
| Akyol 2010 | Pain reduction, improve muscle strength and functional performance | **Short-wave diathermy (SWD):** Induction coil applied circularly along the affected leg. Frequency of 27.12 MHz. Intensity -mild but pleasant sensation of heat. | NR | Inpatient physiotherapy department | 40 mins (20 mins per knee) 3 times a week for 4 weeks | NR | NR | NR | All participants completed treatment |
| Alfredo 2018 | Pain relief | **Laser therapy:** Energy was irradiated over 5 points on the joint line of the medial side of the knee and at 4 points at the lateral side, at 3J per point. Total dose per knee was 27J per treatment.  Wave length of 904 nm, frequency of 700 Hz, average power of 60 mW, peak power of 20W, pulse duration 4.3 ms, 50 seconds per point (area 0.5 cm^2^). | Physiotherapist | Specialist rehabilitation services | 3 times a  week for 3  weeks | NR | NR | NR | NR |
| Alghadir 2014 | Pain relief | **Laser therapy:** Hot packs wrapped in towelling were placed on the target knee(s) for 20 minutes followed by laser therapy delivered via a Ga–As laser device, wavelength of 850 nm, power of 100 mW, and spot size of 1.0 mm. Eight points were irradiated  3 on the medial side of the knee, 3 on the lateral side of the knee, 2 on the medial edge of the tendon of the biceps femoris muscle and semitendinosus muscle in the popliteal  fossa. Each point received energy of 6 J/point for 60s, with a total dose of 48 J/cm2 per session. | Physiotherapist | Physical therapy department | 2 times per  week over 4  weeks | NR | NR | NR | NR |
| Al-Rashoud 2014 | Low-cost, short-application, non-infectious treatment | **Laser therapy:** Energy was irradiated over the joint line at 5 acupuncture points over the medial side of the knee and 4 points on the lateral side, at 1.2J per point for 40 seconds. Total dose per knee was 6 J per treatment. | Physiotherapist | Security forces hospital | 9 sessions | NR | NR | NR | NR |
| Atamaz 2012 | Reduce pain (TENS and IFT), induce an anti-inflammatory response and reduce swelling and pain (SWD) | **TENS:** Frequency of 80 Hz with 10 to 30 mA intensity for 20 minutes. Four surface electrodes were placed over the painful area in the knee region with intensity in the tactile sensation threshold.  **IFT** was applied for 20 minutes with an amplitude-modulated frequency of 100 Hz generated by 4 kHz sinusoidal waves. Two electrodes were placed over the knee region with intensity in the tactile sensation threshold.  **SWD:** continuous SWD with a 10 cm diameter condenser plate operating at a frequency of 27.12 MHz, an input of 300 W, and a mean output of 3.2 W. | Physiotherapist | Physical medicine and rehabilitation dept | 5 times a week for 3 weeks | NR | NR | NR | NR |
| Cakir 2014 | Pain relief | **Continuous US:**, 1 MHz, 1 W/cm^2^, 5cm^2^ head over the painful area in the knee region.  **Pulsed US:** same parameters except a 1:4 pulse ratio was used. | Physiotherapist | Physical medicine and rehabilitation dept | 5 times a week for 2 weeks, approx 12 minutes per session | NR | NR | NR | NR |
| Carlos 2012 | Treatment of pain and loss of function | **Continuous US:** 1 MHz, average power output 7 W, 1.5 W/cm^2^ (100%), 5 minutes to the medial side and 5 minutes to the lateral side of the knee.  **Pulsed US:** 1 MHz, average power output 7 W, 2.5 W/cm^2^, pulsed mode (20%), frequency of 100 Hz. | Physiotherapist | NR | 10 minutes, 3 times a week for 4 weeks | NR | NR | NR | NR |
| Cetin 2008 | Pain control | **Hot pack** (Group 4) provided prior to exercise. No other detail was provided. | NR | Department of Physical Medicine and Rehabilitation at a university | 3 times a week for 8 weeks | NR | NR | Compliance was calculated as the number of patients who completed treatment by the number of initial patients. | Compliance of 85% in the hot pack group. |
| Cheing 2004 | Pain relief | **TENS:** 140 µs pulses at 80 Hz for 60 minutes. Four surface electrodes were placed on acupuncture points. Intensity was adjusted to produce a tingling sensation approximately 3 to 4 times the patient’s sensory threshold. Each participant received the treatment around the same time of the day throughout the treatment period, to avoid fluctuation of pain intensity during the day. To blind the participants from the placebo effect, all participants were told that they might or might not feel the stimulation. 20 minutes of rest was provided before exercise. | NR | Hospital | 20 sessions (5 days a week over 4 weeks) | NR | NR | NR | NR |
| Chen 2014 | Treatment of popliteal cyamella in OA | **Shockwave therapy**: Impulse energy flux density of 0.03 to 0.4 mJ/mm^2^ (scaling from 1 to 20), a frequency of 1- 8 Hz and a pressure range of 11-82 MPa, 2000 impulses for each popliteal cyamella. Dose was applied according to the general therapeutic dose for calcific tendinopathy and the level of density depended on the size of the popliteal cyamella (greater density for larger cyamella). Average intensity was 15-18 scales.  **Pulsed US:** Frequency of 1 MHz and a spatial and temporal peak intensity of 2.5 W/cm^2^, duty cycle of 25%. The probe was applied for 10 minutes to the popliteal cyamella as indicated by real-time 5-12 MHz high-resolution linear scanner and X-ray image of bilateral knees followed by tender point findings made during orthopaedic examination: around 10 cm^2^ in the total treated area. Intensity adjusted so that patients felt a warm sensation or mild sting. | NR | NR | **Shockwave:** Once weekly for 6 weeks ( weeks 1-6)  **US:** three times a week for 8 weeks. | NR | NR | NR | Compliance (number of participants who completed the treatment course divided by the number of initial participants) as 93.3% in the US group and Shockwave groups. |
| De Matos Brunelli Braghin 2018 | Reduce pain, improve physical function and improve cartilage regeneration | **Laser therapy:** Wavelength of 808 nm, 0.028 cm^2^ spot area, 100 mW power, fluence of 200 J/cm^2^, energy of 5.6J per point over tibial and femoral lateral and medial epicondyle, medial and lateral joint lines, and popliteal fossa, biceps femoris, semitendinosus tendons and between the tendons and on the patellar tendon region. Total of 10 isolated points, for 56 seconds per point, total energy of 56 J. | Physiotherapist | NR | Twice a week for 2 months (total of 15 sessions) | NR | NR | NR | Intervention was completed by 6-/71% of the volunteers |
| de Paula Gomes 2018 | Relieve pain and improve function | **Laser Therapy:** Portable 9-diode cluster device with one 905 nm super pulsed diode laser (peak power: 8.5 W; frequency: 1000 Hz; mean power of each diode: 0.9 mW), four 875 nm LED (mean power of each diode: 17.5 mW) and four 640 nm LED (mean power of each diode: 15 mW). Treatment of 3 quadrants overlapping of the medial, lateral and posterior knee in random sequence for 1 minute in each quadrant. Energy per quadrant was 7.85 J, generating a total energy of 23.55J per session. | Physiotherapists | Physical therapy clinic | Twice a week over 5 weeks (10 sessions) of 10 minutes of laser therapy | NR | NR |  | One participant discontinued treatment. Mean number of treatments was 9.6 (SD=1.78) in the laser +ex group. |
| Elboim-Gabyzon 2013 | Increase quadriceps muscle strength | **NMES** Two self-adhesive  electrodes were placed over rectus femoris proximal muscle belly and vastus medialis muscle belly. The patient was seated on a straight-backed chair, with hips flexed at 90° and knees flexed as close to 90° as could be tolerated. NMES parameters: biphasic waveform, PD = 200\|s; frequency = 75 Hz; ramp up 2s; on time 10s; off time 50s; current amplitude to tolerance (max 100 mA). Number of contractions = 10. | Physical Therapists | Orthopaedic outpatient physical therapy clinic | Twice a week for 6 weeks (12 sessions) for 45 minutes per session | NR | NR | NR | A total of 13 of the 63 (21%) patients recruited (all groups) did not complete the treatment protocol due to non-compliance, medical conditions or inability to tolerate electrical stimulation |
| Guidini Lima 2022 | Analgesic effects | **Laser Therapy:** participants received 9 irradiation points in the knee joint, 5 in the medial synovial region and 4 points in the lateral synovial region. Each point was irradiated for 30s and received a total of 3 J, continuous mode with a fixed power of 100 mW. | NR | Physiotherapy outpatient clinic | 3 times a week, on alternate days, for 6 weeks, after performing the exercises | NR | NR | NR | NR |
| Gunaydin 2020 | Pain relief | **Shockwave Therapy:** Affected knee was flexed at 90°. The intervention area on the tibiofemoral and patellofemoral joints was identified with a pen. The probe was then placed on the marked area. An average  of 2000 beats at a frequency of 6–8 Hz. was used per session. Peroneal nerve and vein structures were avoided. | Physiotherapist | School of Physiotherapy and Rehabilitation in University | Once a week for 6 weeks. | NR | NR | NR | NR |
| Gur 2003 | Pain reduction | **Laser Therapy:** applied to 2 points at anterolateral and anteromedial  portals of the knee. The anterolateral portal was located 1 cm above the lateral joint line and 1 cm lateral to the margin of the patellar tendon. The anteromedial portal was located 1 cm above the medial joint line and 1 cm above the edge of the patellar tendon. In Group I, 5-minute stimulation time, 200-nanosecond maximum pulse duration, 2.5 kHz pulse frequency, 20W maximum output per pulse, 10 mW average power, 1 cm^2^ surface, 3J total energy and 30 J accumulated dose were applied. In Group II, 3-minute stimulation time, 200 nanosecond maximum pulse duration, 2.8 kHz pulse frequency, 20W maximum output per pulse, 11.2 mW average power, 1 cm^2^ surface, 2J total energy and 20 J accumulated dose. | Physiotherapist | NR | 10 treatments over 2 weeks | NR | NR | NR | NR |
| Hammami 2021 | Increase muscle strength | **NMES:** carrier frequency modulated at 50 Hz to deliver a biphasic pulse every 200µs and at constant square-wave voltage). Intensity was set at approximately 80% of each participant’s maximum tolerance.  The quadriceps was electrically stimulated during the extension phase and the hamstrings were stimulated during the flexion phase, thereby in eccentric mode and in phase with the eccentric isokinetic contraction. | NR | Department of Physical Medicine and Rehabilitation at orthopaedic institute | Twice a week separated by at least 48 h for 6 weeks. sets-repetitions number was 2 x 10 for weeks 1–2, 3 10 for weeks 3–4 and 4 10 for weeks 5–6 with 1 min recovery  between sets | NR | NR | NR | NR |
| Imoto 2013 | Increase muscle strength | **NMES:** patient seated with hip and knee in 90° flexion and instructed to perform a quads contraction when NMES was received. NMES was applied via 2 self-adhesive electrodes positioned over rectus femoris and vastus medialis via a pulsed rectangular symmetrical biphasic current: frequency 50 Hz, pulse duration 250µseconds, on:off time 10:30 seconds. Intensity was maximum tolerated by the patient. | NR | Speciality ambulatory clinic at a secondary care facility | Twice a week, for 8 weeks for 20 mins | NR | NR | NR | All patients received allocated treatment |
| Jorge 2022 | Incremental therapeutic benefits in pain and physical function | **Laser:** wave length of 808nm, maximum output power of 100mW ± 20%, continuous mode, laser beam spot size of 0.03 cm2, power density of 3.33W/cm2. Four points on the medial and lateral aspect of the knee perpendicular to the knee joint line at 6 JPP (total energy 48 J per session). | Physiotherapist | University clinic | 3 times per week, duration not reported | NR | NR | NR | Participants attended 17 of the 24 planned treatments on average. Drop-out rate in the active laser group was 18%, and in the placebo group was 14% at end of treatment (8 weeks) |
| Kapci Yildiz 2015 | Analgesic effects (continuous via thermal effects and pulsed via non-thermal effects) | **Continuous US**: frequency: 1 MHz, intensity: 1.5 W/cm2, duration: 5 minutes.  **Pulsed US**: frequency: 1 MHz, intensity: 1.5 W/cm2, mode: 1/5, duration: 5 minutes. | Physiotherapist | NR | 5 days a week for 2  weeks | NR | NR | NR | NR |
| Karadag 2019 | Reduce pain and stiffness | **Heat therapy:** Patients were given 2 hot-packs to be applied to both knees and were recommended to use them for 20 minutes twice a day for 5 days a week. | Physiotherapist | Hospital-based physical therapy and rehabilitation polyclinic | Twice a day for 5 days a week. | NR | NR | All patients in the intervention group were called three times a  week and asked if they performed the practices, and the patients  who did not perform the practices regularly were excluded from the sample group. | NR |
| Karakas 2020 | Reduce pain, improve physical function and improve cartilage regeneration | **Pulsed US (**1 Mhz, 1w/cm², 1:4 ratio) was administered with patients in supine and knees in flexion to  cover the knee joint, medial and lateral joint spacing, and suprapatellar regions. average local exposure  time was 1 minute, and the effective radiating area of the transducer head was 5 cm² | Researcher | Department of Physical Medicine  and Rehabilitation at university | 3 sessions a week for 8 weeks (24 sessions) for 10 minutes | NR | NR | NR | NR |
| Kheshie 2014 | Pain relief | **Laser Therapy**: HILT was applied for 15 minutes per session using a pulsed laser, transversely and longitudinally in the anterior, medial and lateral aspects of the knee joint with emphasis on the joint line between the tibial and femoral epicondyles. Total energy delivered during one session was 1,250J through three treatment phases (phase 1- 710 and 810 mJ/cm^2^ for total of 500 J. In the intermediate phase, the handpiece was applied on the joint line just proximal to the medial and lateral tibial condyles with 25J, fluency of 610 mJ/cm2, and 14 s for each point and a total of 250 J in this phase. The final phase was the same as the initial phase except that slow manual scanning was applied. The application time for all 3 phases was approximately 15 minutes with the total energy delivered during one session of 1250 J.  **LLLT:** A Gallium-arsenide diode laser infrared probe with a wavelength of 830 nm, output power of 800 mW, average energy density of 50 J/cm^2^, frequency of 1 KHz and duty cycle of 80%. Patients were in supine lying while the affected knee was slightly flexed and supported with a pillow. The cluster laser was in direct contact and perpendicular to the affected knee 32 minutes & 33s per session, total energy of 1250 J. | NR | Physical therapy dept at university | 2 sessions/ week for 6 weeks (12 treatments). Treatment session duration was 15 mins | NR | NR | NR | NR |
| Kholvadia 2019 | Pain relief | **LLLT:** Participants were exposed to 3 different arrays as part of the LLLT protocol over 12 sessions, with each session progressing from 35-45 minutes. The participant’s knee was set at 60-70°. A circumferential application method was employed. Three placements with medial and lateral applications overlapping at the patella’s surface were used. | NR | Biokinetics rehabilitation centre | Three times a week for 4 weeks (12 weeks) | NR | NR | NR | NR |
| Ones 2006 | Facilitates tendon extensibility and muscle relaxation | **Heat therapy:** hot packs wrapped in towels applied for 20 minutes with patients in supine, followed by deep heating with continuous US (1 MHz frequency, 1.5 W/cm2) using a 4 cm diameter applicator. US therapy lasted for 5 minutes to each knee in each session. | Physical Therapist | Outpatient physical medicine and rehabilitation centre | 25 mins, daily over 15 days (15 sessions) | NR | NR | NR | All patients completed the treatment |
| Park 2021 | Increase muscle strength | **NMES** was delivered via a EMS suit. The exercise regimen consisted of abdomen crunches, bridge, leg raises, side planks, back extension, front planks, front lunges, and squats for 6 s with a 4-s break in between electrical stimulations. The EMS suits enabled the simultaneous activation of eight pairs of muscle groups (both upper legs, both upper arms, buttocks, abdomen, chest, lower back, upper back, and latissimus dorsi) with selectable intensities for each region, and the electrical strength of the suit was controlled via Bluetooth. Stimulation frequency was set at 85 Hz, impulse width at 350 ms. The percentage of maximal tolerance was obtained through the rating of perceived exertion (RPE) scale ranging from 6 to 20- 60% of 1 RM from baseline to week 2, 70% from week 3-5, and 80% from week 6-8. |  |  | 30 minutes, three times a week on two non-consecutive days ( | The EMS  suits comprised silicone conductive pads and wireless materials, were tailored to the participants’ body size. | NR | NR | NR |
| Pietrosimone 2011 | Increase voluntary quadriceps activation | **TENS:** Continuous TENS biphasic pulsatile current at 150 Hz, with a phase duration of 150 microseconds. Four separate self-adhesive electrodes were applied on the medial and lateral superior, and medial and lateral inferior, borders of the patella. Care was taken not to place TENS electrodes on the quadriceps muscles or muscles of the anterior leg. The 2 pairs of electrodes were crossed to encompass the most surface area under stimulation. Participants were instructed to utilise the TENS units during all therapeutic exercise sessions and at least 8 hours per day when they were the most active. Participants were educated on the respective TENS unit operation and electrode application. A daily log was utilised to track compliance of treatment duration. Participants were instructed on how to increase and decrease amplitude, which could be adjusted between 1 and 60 mA. Amplitude was set at a strong, comfortable intensity that was not strong enough to elicit muscle contraction. Participants were instructed to maintain this sensation throughout each treatment session by adjusting intensity as needed. Participants were instructed to maintain the intensity at a level above 5 out of 100 throughout the day. | Certified athletic trainer or licensed physical therapist | University health system | Home use for at least 8 hours a day when patients were most active and during all therapeutic exercises over 4 weeks | NR | NR | A daily log was used to track treatment compliance | Compliance data was not completed by one person in the placebo group. TENS group reported longer intervention use during days 1-7 and days 22-28. No statistical difference in overall time between the 2 groups. |
| Pietrosimone 2020 | Analgesic effects | **TENS**: identical TENS and four separate reusable self-adhesive electrodes were used to deliver TENS. An unblinded investigator, who was not involved in collection of outcome measures, instructed each participant on how to  properly apply the electrodes on the knee joint and operate the TENS unit. Electrodes were applied on the medial and lateral superior and inferior borders of the patella. Electrodes were positioned close to the patella and away from the quadriceps and musculature of the anterior leg. Participants were instructed to use the TENS during all exercise sessions and during activities of daily living. Frequency was set at 150 Hz, pulse duration of 150 µs. Participants could adjust the amplitude between 1-60 mA and were instructed to adjust the amplitude to a strong, manageable sensory stimulation intensity that was not strong enough to elicit muscle contraction and to maintain this sensation throughout each treatment by adjusting intensity as needed. | Licensed physical therapist | Single centre physical therapy clinic | 10 sessions over 4 weeks. Participants were also advised to use TENS throughout activities of the daily loving over the 4 weeks. | NR | NR | NR | NR |
| Quirk 1985 |  | **IFT** was applied using suction electrodes for 10 minutes in a 0 Hz-11 Hz rhythmical frequency, followed by 5 minutes at 130 Hz.  **SWD** was administered for 20 minutes using the condenser field technique. | Physiotherapist | Physiotherapy Dept | 3 times a week for 4 weeks | NR | NR | NR | NR |
| Raeissadat 2018 | Increase muscle strength | **EMG Biofeedback:** A single-channel MyoTrac Infiniti Continence Suite device was used. Skin was shaved and ethanol applied to decrease skin impedance. Gel-contained electrodes were attached according to the SENIAM protocol. Active and reference electrodes were attached to the VMO muscle. The active electrode was attached 4 cm superiorly and the reference electrode 3 cm medially to the superomedial aspect of the patella. The ground electrode was attached to the ipsilateral leg 2-3 cm inferior to the patella. Patients lay supine with a rolled towel approximately 10 cm placed under the knee, and he/she was asked to press the towel as hard as possible for 5 seconds, relax for 10 seconds, and repeat 3 times so that the device could detect and record 3 values of maximum voluntary activity of the muscle and calculate the mean. The voluntary activity threshold of the patient was set at 20% less than the calculated mean. Each time patients managed to contract their muscles beyond the established threshold, the device gave them positive feedback (puzzle getting completed one piece at a time or an animated car moving across the monitor). The physician increased the threshold at each session, according to the patients’ strength, to encourage increased muscle activity. | Specialist | Physical medicine and rehabilitation ward in a hospital | 12 x 15-minute sessions over 2 months | NR | NR | NR | NR |
| Rattanachaiyanont 2008 | Pain reduction and improvement of function | **Continuous SWD**: The patient lay on a treatment bed with an opaque screen between the bed and a SWD machine to blind the patient. Continuous SWD was delivered using a 10 cm diameter condenser plate at a frequency of 27.12 MHz, input of 300W and a mean output of 3.2W using the condenser plate was wrapped around the affected knee(s). | Physical therapist | Outpatient clinic, rehabilitation medicine department | 20 minutes/ session, 3 sessions/week for 3 weeks | NR | NR | NR | NR |
| Sardim 2020 | Pain reduction | **Laser Therapy/PBM**: At the end of the exercise protocol, in all sessions, PBM was applied to 2 points, using a cluster probe, over the joint anterior line of both knees. The following parameters were used: wavelength 850nm and 670 nm, output power for 850 nm probe was 100 MW and for 670 nm probe was 10 mW, with total output power of 540 mW, energy per point was 30 joules and energy density was 4J/cm^2^. | NR | NR | Twice a week for eight weeks | NR | NR | NR | NR |
| Stausholm 2022 | Pain and inflammation reduction | **Laser Therapy:** Treatment was in adherence to the WALT treatment recommendations per treatment spot: six spots in the medial knee joint line, six spots in the lateral knee joint line, and three spots in the popliteal fossa were irradiated with super-pulsed 904 nm wavelength laser for 50s with a mean intensity of 60 mW, resulting in 3 joules per point (45 joules per knee per session). | Physiotherapist | University outpatient clinic | Three times per week in the first 3 weeks | NR | NR | NR | One person (4%) dropped out of laser group after a few treatments due to family illness |
| Vassao 2020 and 2022* | Modulate inflammatory process associated with OA | **Laser therapy/PBM** was applied on medial and lateral region of the knee affected. A cluster device, with 7 infrared laser beams (wavelength = 808 nm) was used. The irradiation energy was 4J per point for 40 seconds at each location followed WALT recommendations. | Physical therapist | Laboratory of Manual and Physical resource and balance space- Fitness and Health | Twice a week for 8 consecutive weeks. | NR | NR | NR | One patient (7%) from the active group and one from the placebo group (7%) did not attend two consecutive treatment sessions |
| Yilmaz 2010 | Increase exercise compliance and patient motivation | **EMG biofeedback**: patients did the same exercises with EMG biofeedback using a double channel biofeedback machine, attached to the vastus medialis and vastus lateralis respectively. Two active surface electrodes were placed in parallel to muscle fibres and inactive ones were placed equal distance from the active ones. Feedback was given via audio and visual signals. | NR | Outpatient clinic in department of physical medicine and rehabilitation | Three times a week for 3 weeks | NR | NR | NR | All patients completed the exercise programmes |
| Youssef 2016 | Pain -relief | **Laser therapy:**  Group I: Patients supine with the knee slightly flexed and supported on a pillow. Laser was administered with a low power laser (power 50 mW, continuous wave, wavelength 880 nm). Each point received energy of 6 JPP for 60 seconds, with a total dose of 48 J per session.  Group II: Each patient received a LLLT dose of 3 J/cm^2^ with the knee extended. Laser was irradiated over the joint line at 5 points of the synovial region of the medial side of the knee and in 4 points at the lateral side, at 3 JPP. Total dose was 27 J per session. Wavelength of 904 nm, frequency of 700 Hz, average power 60 mW, peak power 20 W, pulse duration 4.3 ms, 50 seconds per point. The parameters followed WALT recommendations for OA. | NR | Outpatient physical therapy clinic | 2 sessions a week for 8 weeks (16 sessions) | NR | NR | NR | NR |

6MWT, Six-minute walk test; CAR, Central Activation Ratio; EMG, Electromyographic; Ex. Exercise G1, Group 1; G2, Group 2; GMed, Gluteus Medius; HILT, High Intensity Laser Therapy; Hz, Hertz; IFT, Interferential Therapy; J, Joules; JPP, Joules Per Point; KOOS, Knee Osteoarthritis Outcome Score; mS, milliseconds; MVIC, Maximum Voluntary Isometric Contraction; nM, Nanometre, NMES, Neuromuscular Electrical Stimulation; NPRS, Numerical Pain Rating Scale; NR, Not Reported; PEME, Pulsed Electromagnetic Energy; ROM, Range of Motion; s, seconds; SENIAM, surface electromyography for non-invasive assessment of muscles; SF-36, Short-Form 36; TENS, Transcutaneous Electrical Nerve Stimulation; TUG, Timed Up and Go; US, Ultrasound; VAS, Visual Analogue Scale; VMO, Vastus Medius Oblique; W, Watts, WALT, World Association of Laser Therapy WOMAC, Western Ontario and McMaster Universities Osteoarthritis Index
