# Supplementary material for: Adjunctive electrophysical therapies used in addition to land-based exercise therapy for osteoarthritis of the hip or knee: A systematic review and meta-analysis
Source: Osteoarthr Cartil Open. 2024 Mar 1;6(2):100457. doi: 10.1016/j.ocarto.2024.100457 (PMC10956074; doi:10.1016/j.ocarto.2024.100457)

**Supplemental File 5: Forest Plots- EPT plus Exercise therapy versus Placebo EPT plus Exercise therapy (physical function outcome)**

1. **Medium-term (<6 months)**


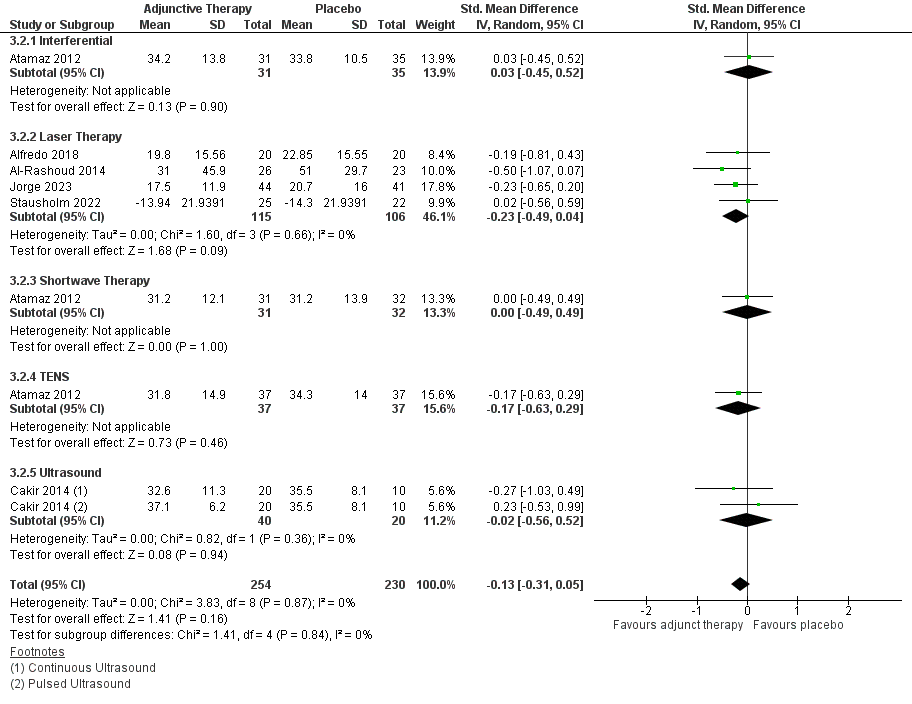


**b) Long-term (> 6 months)**


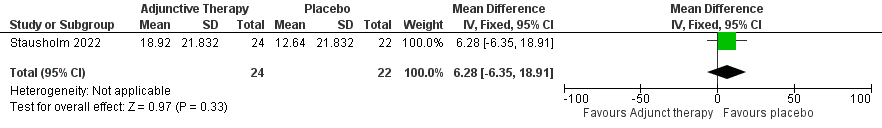

Supplement: Multimedia component 6 [file mmc6.docx]
