# Supplementary material for: Adjunctive electrophysical therapies used in addition to land-based exercise therapy for osteoarthritis of the hip or knee: A systematic review and meta-analysis
Source: Osteoarthr Cartil Open. 2024 Mar 1;6(2):100457. doi: 10.1016/j.ocarto.2024.100457 (PMC10956074; doi:10.1016/j.ocarto.2024.100457)
Supplement: Multimedia component 9 [file mmc9.docx]

**Supplemental File 8: Forest Plots- EPT plus Exercise therapy versus Placebo EPT plus Exercise therapy (Adverse Events)**

1. **Short-term**

**
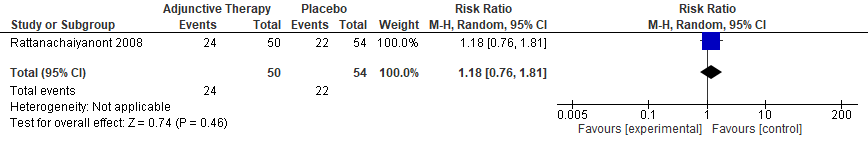
**
