# Supplementary material for: Adjunctive electrophysical therapies used in addition to land-based exercise therapy for osteoarthritis of the hip or knee: A systematic review and meta-analysis
Source: Osteoarthr Cartil Open. 2024 Mar 1;6(2):100457. doi: 10.1016/j.ocarto.2024.100457 (PMC10956074; doi:10.1016/j.ocarto.2024.100457)

**Supplemental File 9: Forest Plots- EPT plus Exercise therapy versus Exercise therapy (Pain and Physical Function and Quality of Life)**

1. **Pain (medium-term)**

**
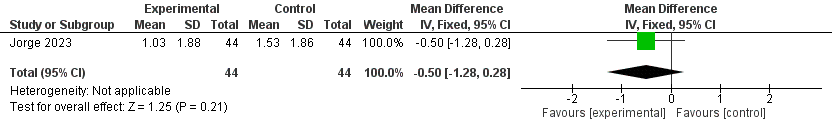
**

**(b) Physical Function (medium-term)**

**
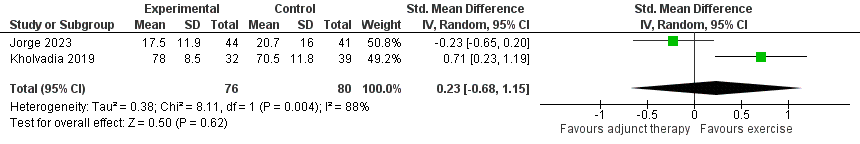
**

**(c) Quality of Life (short-term)**

**
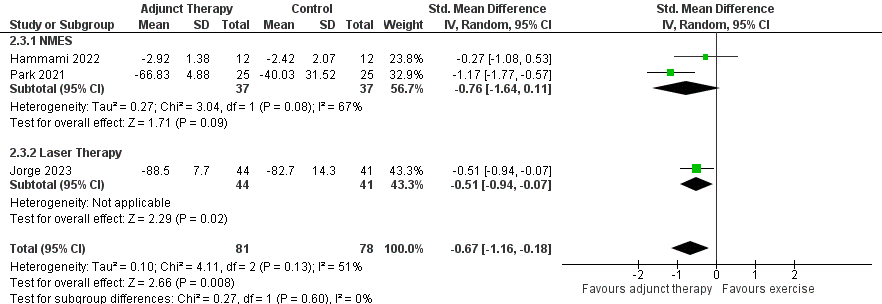
**

**(d) Quality of Life (medium-term)**


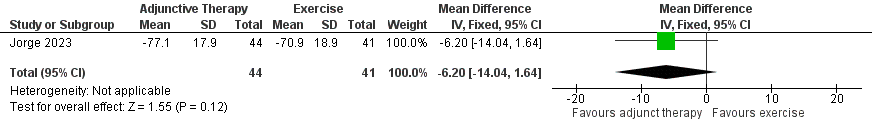

Supplement: Multimedia component 10 [file mmc10.docx]
