# Supplementary material for: Adjunctive electrophysical therapies used in addition to land-based exercise therapy for osteoarthritis of the hip or knee: A systematic review and meta-analysis
Source: Osteoarthr Cartil Open. 2024 Mar 1;6(2):100457. doi: 10.1016/j.ocarto.2024.100457 (PMC10956074; doi:10.1016/j.ocarto.2024.100457)
Supplement: Multimedia component 11 [file mmc11.docx]

**Supplemental File 10: Funnel Plots**


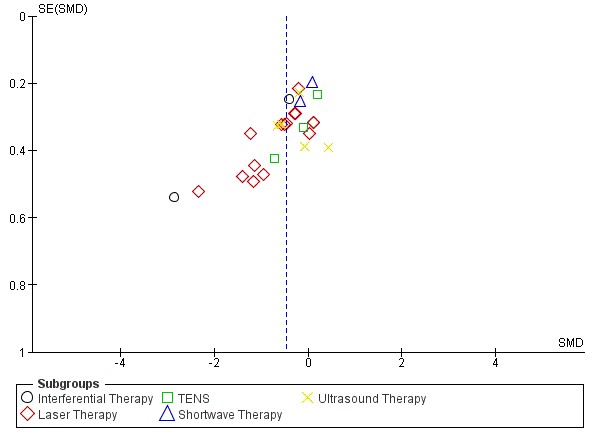
Su

Supplemental Figure 10a: Funnel plot of EPT plus Exercise therapy versus Placebo EPT plus Exercise therapy: pain outcome (short-term)


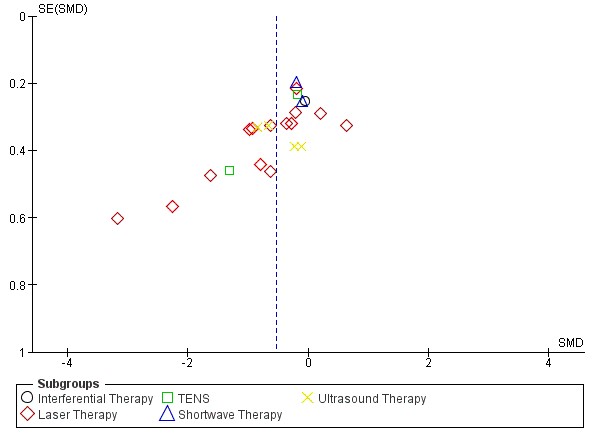


Supplemental Figure 10b: Funnel plot of EPT plus Exercise therapy versus Placebo EPT plus Exercise therapy: physical function outcome (short-term)


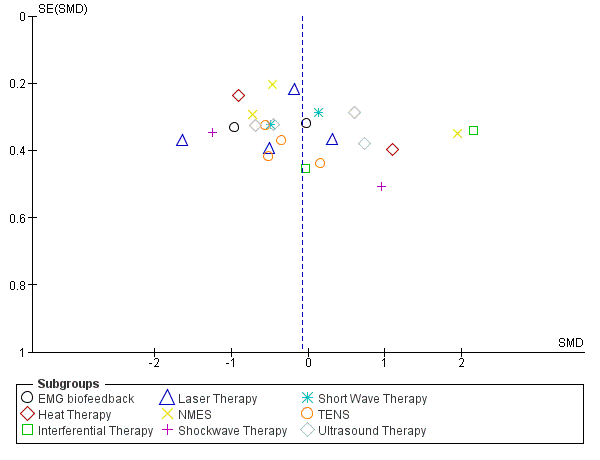


Supplemental Figure 9c: Funnel plot of EPT plus Exercise therapy versus Exercise therapy: pain outcome (short-term)


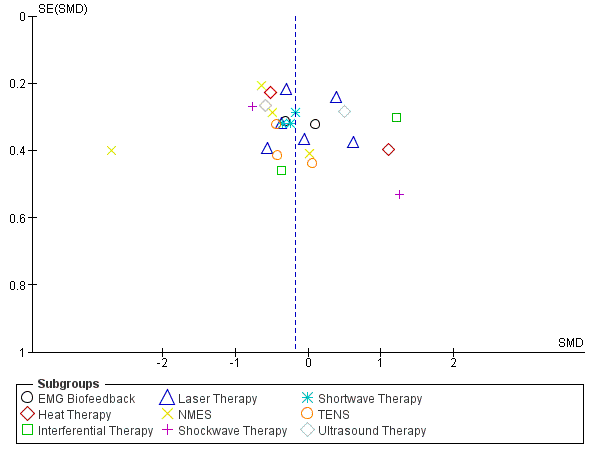


Supplemental Figure 9d: Funnel plot of EPT plus Exercise therapy versus Exercise therapy: physical function outcome (short-term)
